# Determining critical periods for thermal acclimatisation using a distributed lag non‐linear modelling approach

**DOI:** 10.1002/ece3.11451

**Published:** 2024-05-31

**Authors:** Matteo Redana, Chris Gibbins, Lesley T. Lancaster

**Affiliations:** ^1^ Department of Zoology University of Cambridge Cambridge UK; ^2^ School of Environmental and Geographical Sciences University of Nottingham Malaysia Semenyih Malaysia; ^3^ School of Biological Sciences University of Aberdeen Aberdeen UK

**Keywords:** CT_max_, distributed lag non‐linear models, ectotherms, thermal acclimation, thermal history

## Abstract

Rapid changes in thermal environments are threatening many species worldwide. Thermal acclimatisation may partially buffer species from the impacts of these changes, but currently, the knowledge about the temporal dynamics of acclimatisation remains limited. Moreover, acclimatisation phenotypes are typically determined in laboratory conditions that lack the variability and stochasticity that characterise the natural environment. Through a distributed lag non‐linear model (DLNM), we use field data to assess how the timing and magnitude of past thermal exposures influence thermal tolerance. We apply the model to two Scottish freshwater Ephemeroptera species living in natural thermal conditions. Model results provide evidence that rapid heat hardening effects are dramatic and reflect high rates of change in temperatures experienced over recent hours to days. In contrast, temperature change magnitude impacted acclimatisation over the course of weeks but had no impact on short‐term responses. Our results also indicate that individuals may de‐acclimatise their heat tolerance in response to cooler environments. Based on the novel insights provided by this powerful modelling approach, we recommend its wider uptake among thermal physiologists to facilitate more nuanced insights in natural contexts, with the additional benefit of providing evidence needed to improve the design of laboratory experiments.

## Graphical abstract

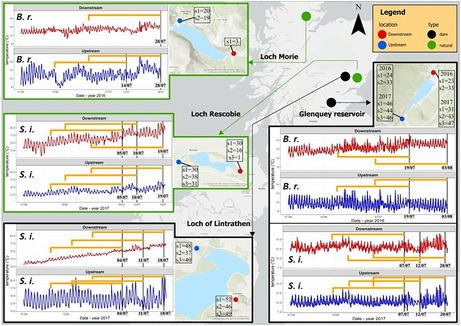

## 1 INTRODUCTION

Thermal environments are undergoing rapid changes, with global warming, human infrastructure, urbanisation and land use changes all modifying thermal landscapes (IPCC, 2014). Under these modified conditions, many species and populations may face temperatures close to or exceeding the limits of their tolerance (Gunderson & Stillman, 2015; Sunday et al., 2014). While all taxa are vulnerable to warming on a global scale (Román‐Palacios & Wiens, 2020), dispersal‐limited species that have reduced ability to escape from patches of extreme temperature are especially vulnerable to localised thermal modification. Thermal stresses are evident in all biomes and habitats, including terrestrial (Lancaster & Humphreys, 2020), freshwater (Johnson et al., 2024) and marine (Oliver et al., 2018) systems. Moreover, thermal threats to populations may reflect not only long‐term climate trends but also localised or more immediate fluctuations in temperature reflecting acute heatwaves or impacts of microhabitat alteration (e.g. Angilletta et al., 2008; Hall & Warner, 2019; Xiang et al., 2017). These shorter‐term or more localised thermal stressors may pose more significant risks than long‐term climate trends (Ruthrof et al., 2018), and as such it is important to understand the temporal dynamics of organismal responses to locally experienced thermal changes.

Thermal stress can negatively impact multiple aspects of organismal physiology, including disruption of cellular and humeral processes involved in somatic maintenance, development and reproduction, increasing energetic costs, reducing immune function, and in extreme cases, destabilising nucleic acids and proteins, sometimes resulting in death (Williams et al., 2016). Thermal acclimatisation is one way that species may adaptively respond to thermal stress, by using local thermal cues to develop protective phenotypes (Lancaster & Humphreys, 2020; Somero, 2010). Thermal acclimatisation is widespread (observed in all domains of life) and represents adaptive modification of an individual's thermotolerance based on its recent or long‐term experience of environmental temperatures (Schulte et al., 2011), and is usually accomplished via upregulation of protective molecules and shifts in physiological allocation (González‐Tokman et al., 2020). Thermal acclimatisation may help facilitate survival under novel, anthropogenic environmental thermal regimes (Gunderson & Stillman, 2015), if these regimes are able to induce adaptive responses. Moreover, individuals are particularly vulnerable to novel thermal extremes when not acclimatised (Lancaster & Humphreys, 2020; Wood et al., 2018). For these reasons, it is important to understand how natural and human‐altered patterns of thermal variability experienced by organisms in the wild relate to species' acclimatisation responses.

Previous experiments, conducted over a range of ectotherm species, indicate that acclimatisation is influenced in complex ways by an individual's history of thermal exposure; variation in acclimatisation response is influenced by absolute temperatures experienced, timing and duration of exposure and rates of thermal change (Colinet & Hoffmann, 2012; Nyamukondiwa et al., 2018; Smith & Lancaster, 2020; Sullivan et al., 2000). Additionally, the thermal acclimatisation process may involve different physiological mechanisms operating at different timescales. In response to rapid and intense changes in temperature over short timescales, characterised by high rate of temperature variation, rapid thermal hardening effects may be triggered (i.e. via active upregulation of protective molecules such as mucins, heat shock proteins or osmotic regulators) (Bates & Morley, 2020; Moyen, Crane, et al., 2020). However, when new thermal conditions become stable, or even following slower changes in environmental temperature, individuals adapt their physiological state to the new thermal environment (Bates & Morley, 2020). The process of physiological stabilisation operates over longer timescales, such as days or months (Bates & Morley, 2020; Peck et al., 2010), and has usually been measured under laboratory conditions using different indicators, such as long‐term survival (e.g. Peck et al., 2009), stabilisation of metabolic rate (e.g. Robinson & Davison, 2008) and modulation of gene expression (e.g. Banti et al., 2010).

Critical thermal limits (CTLs) are often used to evaluate thermal tolerance. CTLs are defined as the functional boundary temperatures of an organism (Nyamukondiwa & Terblanche, 2010), with the critical thermal maximum (CT_max_) and critical thermal minimum (CT_min_) representing the upper and lower temperature limits of thermal tolerance, respectively. Species' capacities for thermal acclimatisation have often been evaluated as the degree to which alternative thermal exposure histories beneficially alter future CTL expression, both in the laboratory and in the wild. In the laboratory, this has often been assessed using experimental approaches that include a period of temperature equilibrium (Agudelo‐Cantero & Navas, 2019) followed by application of relevant cues such as exposure to a constant modified temperature (Fu et al., 2018; Terblanche et al., 2007) or more rarely to a variable temperature (Auer et al., 2018; Ravaux et al., 2019). In such experiments, shifts in thermal tolerance associated with acclimation, namely the lab‐based proxy for acclimatisation in the wild, are assessed by comparing CTLs of treated and untreated individuals or populations. A large body of previous work suggests, however, that the magnitude of the observed acclimation response (change in CT_max_ or CT_min_) is highly dependent on the methods used for both the acclimation treatment regime (i.e. temperatures, durations or fluctuations applied) and for assessment of thermal tolerance itself (e.g. whether a ramping or static assay is used, and the temperature and duration of any re‐equilibrium period applied between the acclimation treatment and thermal tolerance assessment) (Bates & Morley, 2020; Terblanche et al., 2007). These methodological limitations fail to capture the nuances of field exposure characterised by strong stochasticity and make it difficult to draw firm conclusions about field‐relevant acclimatisation capacity based on laboratory studies.

To increase ecological realism, many researchers have attempted to assess acclimatisation responses in the field. However, organisms in the wild are typically exposed to a variable and stochastic thermal environment, which complicates precise identification of salient acclimatisation cues. In order to quantify the relationship between a wild individual's thermal history and its resulting acclimatisation state, the thermal history is typically summarised in a statistical way to reduce the dimensionality of the data, often using convenient metrics (Rivers‐Moore et al., 2013). Such metrics include thermal thresholds (i.e. temperatures expected to trigger acclimatisation responses) and temporal windows (e.g. instantaneous maximum, 7‐day moving window, etc.) (Simmonds et al., 2019; Sullivan et al., 2000). This approach has proved useful for identifying significant thermal exposures and time windows that determine variation in CTLs (Lancaster et al., 2015). However, it suffers from some of the same limitations as laboratory studies. In particular, decisions about which thresholds and durations to apply will influence the conclusions and, more importantly, statistical summarisation of previous thermal conditions, while analytically tractable, will fail to recreate the organism's real experience; thus, the actual thermal cues for acclimatisation will likely be obscured.

**FIGURE 1:**
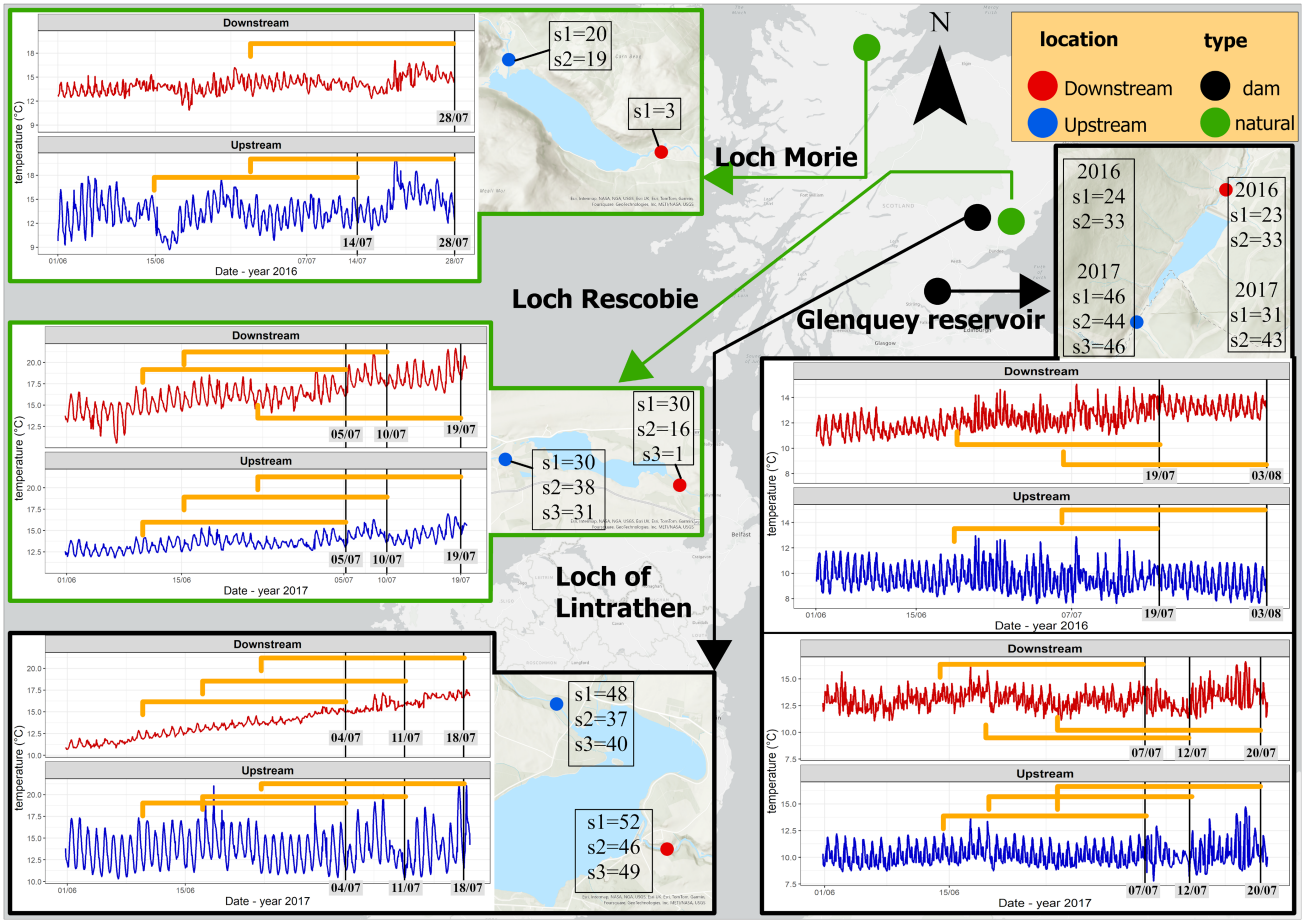
Sampling locations of *Seratella ignita* and *Baetis rhodani* larvae. In the main map, natural lakes are highlighted in green colour, and reservoirs (i.e. dammed lakes) in black. In the zoomed maps are positions of upstream (blue dots) and downstream (red dots) sampling locations for each lake; numbers in boxes are the number of larvae collected for each location on each sampling day (s1–s3) from which the mean CT_max_ has been computed (see Section 2.3). For each sampling location, the water temperature time series is presented, along with the dates of sampling (vertical black lines) and the length of the exposures considered in this study (yellow brackets). In each time‐series box, the label ‘*S.i*.’ refers to *Serratelta ignita* samples and ‘*B. r*.’ to *Baetis rhodani* samples (Basemap: ESRI, 2021).

Modelling the complete thermal history of organisms to extract the salient cues is a promising solution to these issues, but models capable of doing this remain in their infancy. In this paper, we apply and evaluate the potential of distributed lag models (DLMs) in their non‐linear form (DLNMs – Armstrong, 2006; Gasparrini et al., 2010) to improve understanding of how thermal history shapes acclimatisation state. DLNMs are a class of model developed in econometrics and, within the life sciences, are used primarily in medicine and epidemiology (e.g. Almon, 1965; Cui et al., 2020; Dong et al., 2020; Ranjbaran et al., 2020; Schwartz, 2000). They represent an elegant analytical framework to describe associations characterised by a delay between an exposure and a response in time‐series data. For instance, a continuous outcome variable, such as thermal tolerance, can potentially be modelled as a non‐linear function of the predictor variable (here, the individual's complete set of temperature exposures), as well as a continuous set of lag times representing different points in the individual's thermal history at which those exposures were experienced. DLNMs may therefore allow estimation of the effect of an individual's entire thermal history on CT_max_ (Gasparrini et al., 2010), without requiring any a priori assumptions about the thresholds or time periods that might trigger thermal acclimatisation. Specifically, in this paper we develop and apply a novel DLNM analytical pipeline to assess thermal acclimatisation in the wild. As an example, we apply this pipeline to assess the temperatures and their lags that are critical for the short‐term acclimatisation of two common and widespread insects (*Seratella ignita* (Poda 1761) and *Baetis rhodani* (Pictet 1843); Ephemeroptera) in northeast Scotland. These mayfly insects have aquatic larvae, and the thermal environment they are exposed to as larvae can be heavily modified by human activities (Mejia et al., 2020; Seyedhashemi et al., 2021; Stanford & Ward, 2001; Ward & Stanford, 1983). Such anthropogenic modification to river temperatures due to processes such as water abstraction, deforestation or damming is known to affect insect populations (Bruno et al., 2019; Heggenes et al., 2021; Redana et al., 2022).Using a pre‐existing dataset of thermal tolerances of these species in association with short‐term temperature monitoring data from their local river environment, in dammed and non‐dammed Scottish rivers (data were collected by Lancaster, Gibbins and Adela Hrubesova in 2016/17, see Figure 1), we therefore use the opportunity resulting from thermal changes downstream from dams to apply DLNMs to assess acclimatisation of *S. ignita* and *B. rhodani* to river temperature modification.

## 2 MATERIALS AND METHODS

### 2.1 The distributed lag non‐linear model: An overview

The distributed lag non‐linear model (i.e. DLNM) family offers a method to represent non‐linear and delayed dependencies between a response $y_{t}$, measured at time $t_{0}$ and lagged occurrences of the exposure variable $x_{t}$. The exposure variable is expressed as the vector $q_{t}={\left[x_{t-l_{0}},\ldots ,x_{t-L}\right]}^{T}$, where $l_{0}$ and L are the minimum and maximum time lags between an exposure (*x*) and the response ($y_{t}$) at time t (Gasparrini, 2014) within the time space T. The association between the vector $q_{t}$ and the response is modelled in its nonlinear form through the so‐called *cross‐basis* function s, defined as.

(1) $$s\left(q_{t}\right)=s\left(x_{t-l_{0}},\ldots ,x_{t-L}\;\right)=\sum_{l=l_{0}}^{L}f*w\left(x_{t-l},l\right)$$

where $f*w\left(x_{t-l},l\right)$ is a bidimensional function defined as the exposure–lag–response function. It models simultaneously the exposure–response curve $f\left(x\right)$ and the lag–response curve $w\left(l\right)$ along the vector $\;l={\left[l_{0},\ldots ,L\right]}^{T}$. The space $L-l_{0}$ is interpreted as the lag period over which exposure to x is assessed for its influence on the response variable at time t, and thus the thermal history considered. Lag l can take any unit (minutes, hours and days) according to the measurement resolution. The length of the thermal history considered depends on the exposure data availability but should be set according to some ecologically relevant period (e.g. duration of the life stage of interest).

More recently, DLMs and DLNMs have been generalised for application to *longitudinal* data, in which responses are determined at multiple time periods independently for a set of individuals (i.e. datasets containing a range of exposure profiles (*i*), one for each individual, corresponding to different responses, potentially determined at different time periods). The indexing structure i allows each response $y_{i,t}$ to depend on the vector $\;q_{i,t}={\left[x_{i,t-l_{0}},\ldots ,x_{i,t-L}\right]}^{T}$ that is modelled through the longitudinal version of the *cross‐basis* function $s\left(q_{i,t}\right)$. This indexing structure is of particular value as it allows many individuals, with shared or unshared thermal histories, to be modelled together.

The parameterisation of $f\left(x\right)$ and $w\left(l\right)$ is performed through the implementation of the cross‐basis function in a standard regression model such as generalised linear models (GLM), or more recently, in generalised additive models (GAMs) to smooth the exposure–lag–response surface in a penalised context. In addition, the inclusion of the cross‐basis in a GLM or GAM framework allows the inclusion and control of covariate effects that may influence how the exposure history influences the response. Examples considered for thermal acclimatisation may include taxonomy (e.g. Dallas & Rivers‐Moore, 2012; Gunderson & Stillman, 2015; Morley et al., 2019), developmental stage (Agudelo‐Cantero & Navas, 2019), population ancestral temperature range (Lancaster et al., 2015), random effects to control for sampling design or additional covariates for environment and population composition (Wood et al., 2018).

The exposure–lag–response surface can be estimated by predicting the effects of $\beta_{x,l}$ on a grid of predictor values x and l. The estimation is achieved by maximising the model likelihood in terms of the coefficients η of the cross‐basis function and the coefficients of the other covariates included in the model. For ease of interpretation, $\beta_{x,l}$ are defined as specific contrasts of $f*w\left(x_{t-l},l\right)$ by centring the exposure–response function $f\left(x\right)$ to a reference value of the predictor (by default the mean value of the predictor values).

DLNM implementation through the R (R Core Team, 2022b) package *dlnm* (Gasparrini, 2020) requires selection of a basis function to represent $f\left(x\right)$ and $w\left(l\right)$ respectively. Different basis functions are available, including linear, natural splines, b‐splines, linear threshold or piecewise constant (step) functions for non‐penalised DLNM. In the penalised context, the basis functions p‐splines or cubic regression splines are available. From a practical point of view, the selection of the basis function between different splines to model non‐linear relationship should not have major consequences in the estimation of the $\beta_{x,l}$; nevertheless, it has been shown that working under a penalised framework improves DLNM flexibility and inferential properties (Gasparrini et al., 2017).

The package *dlnm* (Gasparrini et al., 2020) further offers a set of default plots to explore $\beta_{x,l}$ predicted by the model; specifically an overall 3D plot (e.g. Figures 4b, 5b) and slice plots (e.g. Figures 4c, 5c) can be produced to evaluate the confidence interval of the predicted effect. In this paper, we add an easy alternative for the visualisation of $\beta_{x,l}$ where the overall 3D plot is represented in two dimensions with the use of a heatmap (e.g. Figures 4a, 5a); in this plot, all the non‐significant combinations of exposure and lag are removed (see Section 2.5 for the interpretation of significant effects in the context of our mayfly example). This 2D visualisation of $\beta_{x,l}$ is based on the package ggplot (Wickham, 2016), and we include this additional plotting code in our pipeline.

Our pipeline for applying DLNMs to the target species works through the processes of data collection, data standardisation, model construction and selection and then interpretation. This pipeline is detailed below. Data are available on Dryad at https://doi.org/10.5061/dryad.4mw6m90gh (Redana et al., 2023), associated code and model implementation tutorials can be found at: https://github.com/monviso/Determining‐Critical‐Periods‐for‐Thermal‐Accliamtion‐Using‐a‐DLNM‐approach.

### 2.2 CT_max_ data collection

We tested the DLNM approach to estimating critical acclimatisation temperatures and lag times for larval blue‐winged olive mayflies, *S. ignita* (Poda 1761), and large‐dark olive mayflies, *B. rhodani* (Pictet 1843), both of which are common and ubiquitous freshwater invertebrates across Europe. Both species usually produce a single generation per year, with eggs overwintering (hatching: ~March/April) and adults emerging from April to September, depending on local thermal conditions (see, López‐Rodríguez et al., 2009; Raddum & Fjellheim, 1993) with non‐overlapping generations in Scotland (Maitland, 1965; Morgan & Egglishaw, 1965). Larvae of both species were collected from four sites in Scotland, in rivers upstream and downstream from two natural lakes (Rescobie Loch and Loch Morie) and two artificial reservoirs created by damming (Loch of Lintrathen and Glenquey Reservoir), giving a total of eight sampling locations (Figure 1).

This combination of sampling locations provided a potentially higher variability in the thermal exposures in terms of absolute temperature, diel variability and trend, allowing us to disentangle salient elements of thermal history that trigger thermal acclimatisation across a variety of background conditions. Sampling locations downstream from dams are considered hereafter as ‘dammed’. Water temperature was continuously recorded at each sampling location with HOBO Pendant UA‐002‐64 data loggers (Onset Corp. Bourne, MA, USA, accuracy ±0.2°C) on a time step of 15 min over the 28 days prior to each larval sample collection. Loggers were placed carefully in locations with running water (riffles) to have good mixing through the water column, and in areas with representative and homogenous morphology of each stream far from sources of potential thermal alteration such as confluences (see Section 4). To facilitate more tractable DLNM modelling times, the temperature data were averaged within each hour of exposure to obtain an average hourly time series. All of the original 15‐min‐interval measures differ by <0.2°C from the average hourly temperature. Due to loggers being occasionally lost due to washout or their malfunctioning, for a small number of days the missing water temperature values of the 28‐day‐long thermal history here considered were estimated through the implementation of generalised additive models (GAMs). For this, GAMs predicting water temperature were trained on a subset of the available data and validated on the remaining subset. Specifically, a set of seven GAMs, separately for each sampling location, were fitted testing as predictors of air temperature at the nearest Met Office station (Met Office, 2012), hour of the day and day of the year, with different implementations. The GAMs were evaluated for each sampling location, by computing the mean absolute error (MAE), MAE standard deviation (MAE SD) and correlation coefficient (*r*). The best models, selected as those with minimum MAE, MAE SD and maximum *r*, had an average MAE = 0.17°C, MAE SD = 0.65°C and *r* = 0.73. The best models were then used to predict water temperature for the missing days. Details of the missing measurement for each sample, the modelling process and the prediction accuracy can be found in the Appendix S1: SM1. In general, the model slightly (~0.7°C) under‐ or overestimates daily maximum and minimum temperatures respectively. To ensure that the error in the predicted temperature did not create biases in the estimation of the exposure effect with DLNM, the same modelling approach we present here in the main paper has been replicated for only samples with measured data available (i.e. using a shorter exposure period and fewer sampling events). We found that the DLNM implemented on the measured‐only water temperature dataset is consistent with the main analysis here reported (see Appendix S1: SM7).

Larval sampling took place within a circle of 5 m radius from the loggers, and within the same riffle, using kick sampling. Subsequent local‐scale spatial mapping of temperatures suggests that within‐riffle temperatures are generally homogenous (M. Redana, L. Lancaster, unpublished data). We considered the temperature recorded representative of the thermal history of the individuals as for the location morphology and hydrology (see above) combined with the relative low mobility of freshwater larvae. Samples were collected on up to three dates at each site (s1–s3 in Figure 1); the species' phenology (see above) ensures that all larvae within each sample were of similar age and developmental stage. Once collected, larvae were placed in containers filled with river water and immediately transported to the laboratory in cooler bags (average trip of 2 h by car). Throughout the transport, we assumed minor variation in the water temperature since the last temperature experienced in the field (see Appendix S1: Table SM1), although in future experiments it is recommended to monitor the thermal conditions during transport. CT_max_ values were established in experiments using high‐performance Grant Optima TX150 circulating water baths with programmable heating settings. Larvae were always handled using plastic spoons to prevent damage. They were placed in eight plastic cups (7 × 15 cm), one for each location. Additional plastic cups were used if the number of individuals exceeded 30. Cups were filled with respective site water in order to preserve thermal conditions and minimise physiological stress. The cups were then placed in the water bath. Prior to experiments, all individuals were equilibrated at 10°C for 10 min. During each thermal trial, samples were subjected to a constant water temperature increase of 0.1°C/min, which was monitored with the water bath, which had the C2G cooling attachment (Grant Instruments, Shepreth, UK). CT_max_ values for each individual were recorded when no physical response occurred after three consecutive prods (Becker & Genoway, 1979); these individuals were then removed from cups while the remaining individuals continued to be monitored. All the CT_max_ experiments were performed on the day of sample collection. No behavioural interactions between individuals were noted during trials.

### 2.3 Response variables: Mean CT_max_

We performed some preliminary analysis on CT_max_ values to identify general patterns. Using a generalised linear model, we established if individual CT_max_ differs (*α* < 0.05) between sites or species, or varies between sampling events at any one site, based on the *t* statistic (see Figure 3). Detailed methods and results are reported in Appendix S1: SM4. Irrespective of the results of these tests, all the data were used to implement the DLNMs. To improve model performance, and because we lacked individual‐level predictors, the response variable in DLNM models is the average CT_max_ ($\bar{{\mathrm{CT}}_{\max}}$), estimated over all the individual larvae collected at each sampling location at each sampling time t (see Figure 1 for the sample size from which each $\bar{{\mathrm{CT}}_{\max}}$ has been computed). Thus, by using $\bar{{\mathrm{CT}}_{\max}}$, we smoothed out the peculiarity of specific individuals, and we can interpret each $\bar{{\mathrm{CT}}_{\max}}$ value as a population‐level average acclimatisation response at time *t* to the complex thermal history of the specific location in which the individuals were captured. To test whether our $\bar{{\mathrm{CT}}_{\max}}$ estimate may be biased by extreme values within time peridos, we tested the leverage effects computing *Z*‐value for each CT_max_ measured, grouped by species, sampling location and date. Individuals with disproportionate influence on the mean (i.e. *Z*‐score > 3 or *Z*‐score < −3) were removed from $\bar{{\mathrm{CT}}_{\max}}$ computation. A total of 13 CT_max_ measures were removed. We further compared model results with and without these extreme individuals, and found highly congruent patterns, suggesting that these outliers had little influence overall. The two reasons for using $\bar{{\mathrm{CT}}_{\max}}$ as a response variable are as follows: (1) within‐sample CT_max_ variability (see Section 3) cannot be attributed to the shared thermal history of the sample site; thus, the use of $\bar{{\mathrm{CT}}_{\max}}$ mitigates the presence of extreme CT_max_ values that potentially could be influenced by alternative and unknown thermal exposures (e.g. recently migrated individuals); (2) the presence of multiple $y_{i,t}$ with different values but sharing the same exposure profile and set of predictors can hinder the model's ability to disentangle water temperature effects within each population at different lags, leading to poorer model performance overall.

Our final dataset contains a total of 25 $\bar{{\mathrm{CT}}_{\max}}$ values (see, Figure 1 vertical line showing sampling time for each location). In this case, we applied DLNM to a longitudinal data structure, where $y_{i,t}$ = $\bar{{\mathrm{CT}}_{\max_{i,t}}}$ with i=1,…,25, for which different exposure profiles (i.e. 25 unique thermal histories) determine the average response within each sampling time. However, individual (i.e. non‐population‐averaged) CT_max_ may be modelled using our approach in cases where individual‐level variability is of interest and individual‐level thermal histories are available.

### 2.4 Exposure variables: Water temperature standardisation

Because short‐term thermal acclimatisation processes may reflect both the magnitude and rate of change in temperature at different timescales (see Section 1), we used both absolute water temperatures and instantaneous rates of temperature change as exposure variables in the DLNMs.

Rate (r) of temperature change (${\mathrm{At}}_{r}$) was computed as

(2) $${\mathrm{At}}_{r_{i}}={\mathrm{At}}_{l_{i}}-{\mathrm{At}}_{l_{i-1}}$$

where ${\mathrm{At}}_{l_{i}}$ and ${\mathrm{At}}_{l_{i-1}}$ are the absolute water temperature at lags i and i−1, respectively (i.e. the difference between the temperature at an hour and the temperature at the preceding hour).

**FIGURE 2:**
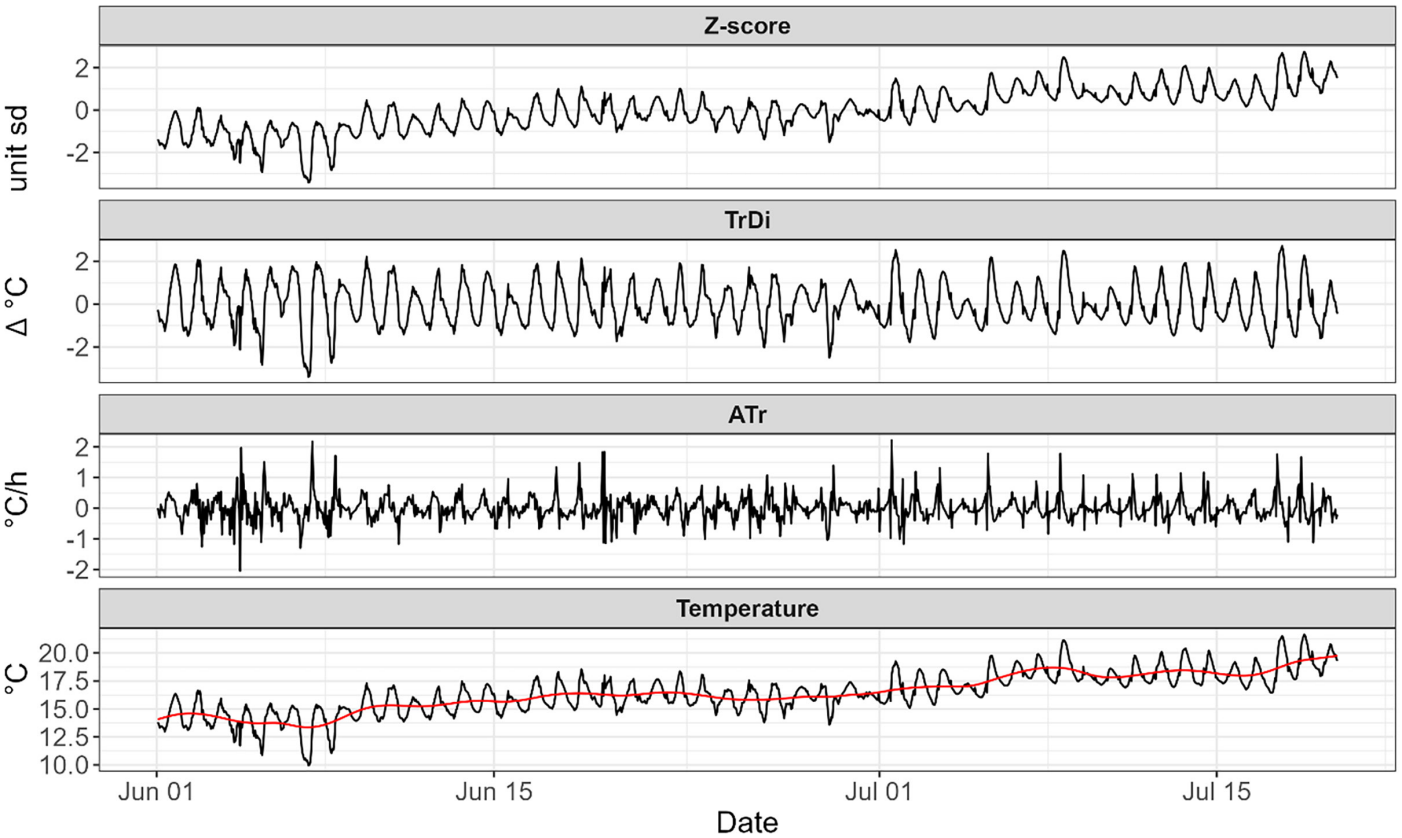
Time series of *Z*‐score, trend distance ($\;{\mathrm{Tr}}_{\mathrm{Di}}$), temperature change rate $\left({\mathrm{At}}_{r}\right)$ and absolute temperature trend. The red line is the modelled smooth trend over which ${\mathrm{Tr}}_{\mathrm{Di}}$ and ${\mathrm{At}}_{r}$ are computed. Standardised water temperature values closer to 0 are water temperatures closer to the mean of the entire period in Zs or the modelled trend in $\;{\mathrm{Tr}}_{\mathrm{Di}}$. Water temperature rate of change *At*_*r*_ closer to 0 (either positive or negative) indicates small differences in temperature between subsequent lags. Here, the time series for Rescobie Loch upstream is depicted as an example plot; in Appendix S1: SM2, standardised values are plotted for all sampling locations.

Each sampling location in our study was characterised by different average absolute temperatures and variabilities (see Section 3.1 and Appendix S1: SM3). Thus, absolute water temperature was standardised in order to control for the different thermal environments (means and variabilities) specific to each sampling location. This standardisation is required to control for longer‐term acclimation or genetic adaptation processes that may differ among sites but are not tractable with our data. For instance, an absolute temperature shift to 12°C might be considered ‘warming’ for a site with a long‐term average of 10°C but would be considered ‘cooling’ for a site with a long‐term average of 13°C. Common standardisation procedures, however, such as *Z*‐score, based on the mean and standard deviation computed over the whole set of data, can suffer a temporal bias by standardising all values to an arbitrary reference temperature, the deviations from which may not be equally salient to organisms at all exposure times. For instance, warming events (*z*‐score > 0) early in the exposure history may magnify or diminish further acclimatisation response to temperatures with similar *z*‐scores occurring later in the exposure history. Deviation from the overall temperature trend may therefore represent a more appropriate standardisation. We compared these two methods of standardisation using our temperature data. The temperature trend distance method to standardise absolute temperature (${\mathrm{Tr}}_{\mathrm{Di}}$) was computed as:

(3) $${\mathrm{Tr}}_{{\mathrm{Di}}_{i}}={\mathrm{At}}_{l_{i}}-{\mathrm{Tr}}_{l_{i}}$$

where Tr is water temperature trend computed using generalised additive model (GAM) in the form of $\mathrm{At}=f\left(\text{time}\right)$, where $f\left(\text{time}\right)$ is implemented with a cubic regression spline with number of knots chosen for each sampling location to smooth the between‐day temperature trend in the period but exclude the within‐day temperature variation. In Appendix S1, we provide full set of exposure values produced by these two methods for all the sites, along with summary statistics. As an example, in Figure 2, we show ${\mathrm{At}}_{r}$, ${\mathrm{Tr}}_{\mathrm{Di}}$ and Zs for one of the sites (Rescobie Loch upstream). The interpretation of Zs is straightforward; values have been centred on the mean temperature over the time period, with variance expressed in standard deviation units. For ${\mathrm{Tr}}_{\mathrm{Di}}$, positive values represent temperature exposures at lag l greater than the modelled trend Tr at that lag l, while negative ${\mathrm{Tr}}_{\mathrm{Di}}$ values are temperatures lower than Tr at that lag l; the closer ${\mathrm{Tr}}_{\mathrm{Di}}$ are to 0 the closer the temperature at that lag l is to Tr. Finally, for ${\mathrm{At}}_{r}$, the closer values are to 0, the smaller the rate of change of the absolute temperature between two consecutive lags; conversely, the higher the absolute ${\mathrm{At}}_{r}$ (i.e. extremely positive or extremely negative), the faster rate of water temperature change (Figure 2).

### 2.5 DLNM implementation

**FIGURE 3:**
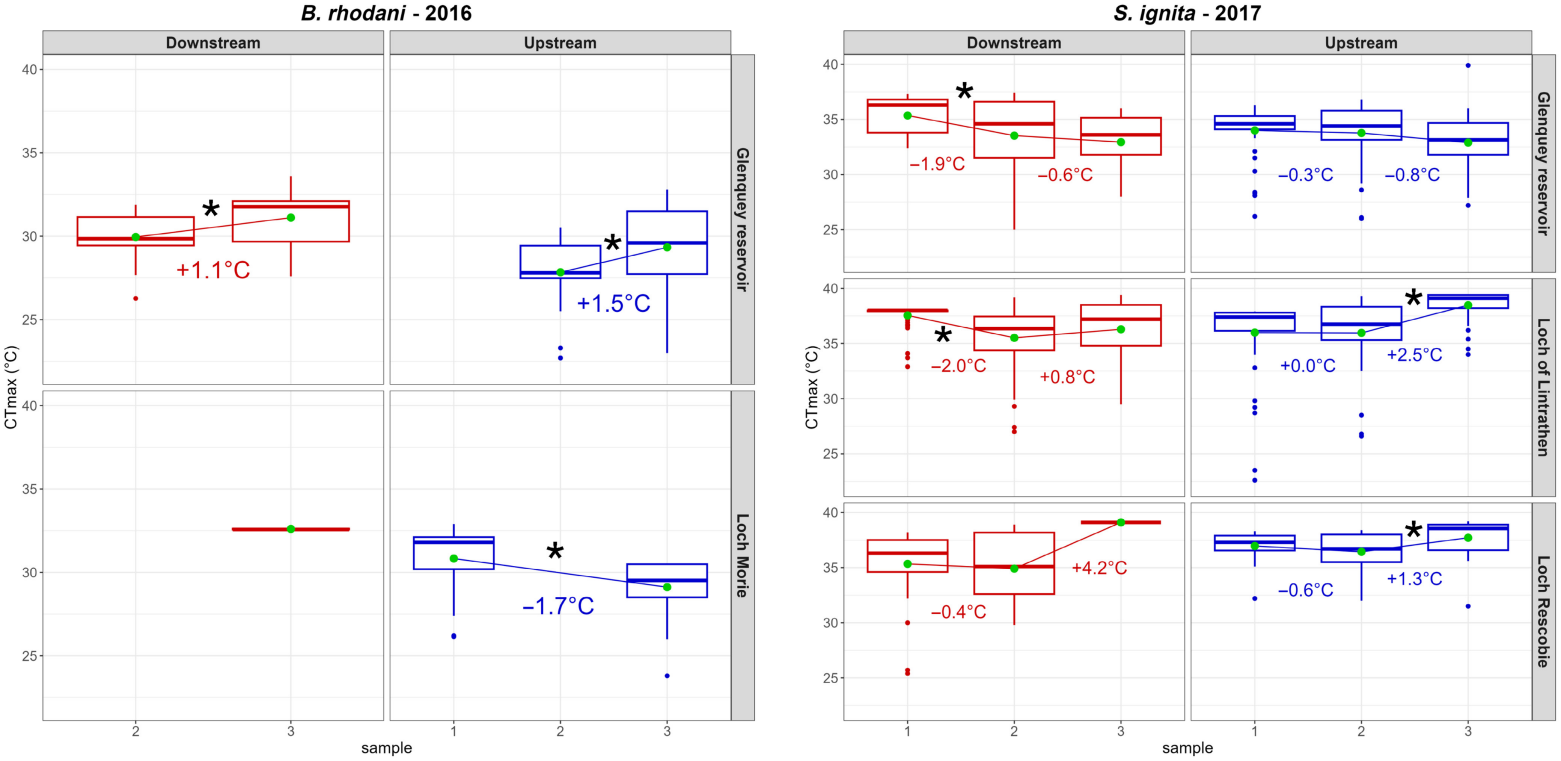
Boxplot summary of CT_max_ values for each site and species. Horizontal lines indicate 25th, 50th (median) and 75th percentiles. Green dots depict the $\bar{{\mathrm{CT}}_{\max}}$. Connecting lines and associated numbers indicate the direction and magnitude of change between respective dates, with ‘*’ indicating significant differences in $\bar{{\mathrm{CT}}_{\max}}\;\mathrm{at}\;\alpha =.05$.

To assess a general response, we implemented DLNM for both species combined, using covariates to account for species‐level variation. Our DLNMs have the general form

(4) $$E\left(y_{i,t}\right)=\mathrm{cb}+u_{\mathrm{sl}}+\mathrm{sp}$$

where $u_{\mathrm{sl}}$ is sampling location as a random effect and sp is the fixed effect of species on $\bar{{\mathrm{CT}}_{\max}}$ (Figure 3). We initially included other potential factors such as downstream/upstream and dammed/natural rivers, but these were removed from the final model due to the fact that they did not significantly impact variation in $\bar{{\mathrm{CT}}_{\max}}$ or improve model fit (Figure 3). However, to assess whether responses for individual species may not be captured by the combined model, we also separately modelled the response of *S. ignita*, the most abundant of the two taxa. We found that the effects of exposure on $\bar{{\mathrm{CT}}_{\max}}$ variation were similar in shape and magnitude to the combined‐species models presented here; however, the reduced number of observations substantially increased the estimation uncertainty (no lag‐exposure combinations were significant, except marginally for the *At*_*r*_‐based model). We report the full analysis of *S. ignita* in Appendix S1.

Following Gasparrini et al. (2017), we compute the DLNMs under a penalised framework implementing the cross‐basis function in a GAM due to its better inferential performance compared to unpenalised methods (i.e. generalised linear models). The cross‐basis function (cb) is modelled via Equation 1. For each water temperature standardisation method, we fitted a set of DLNMs testing different combinations of:

1. Linear and non‐linear basis functions (specifically cubic splines)
2. Inclusion–exclusion of the intercept on the exposure dimension at $l_{0}$ (i.e. the exclusion of the intercept at $l_{0}$ forces exposures closer to the sampling time t to have no effect on CT_max_, so that $f*w\left(x_{t-l},l_{0}\;\right)=0$) for each standardisation method.

The ecological implication of the cross‐basis parameterisation is considered further in the Section 4. For each model, we computed the AIC score and the deviance $\bar{{\mathrm{CT}}_{\max}}$ explained (Wood, 2017). We estimated the exposure–lag–response surface for all combinations of lags, with L = 673 (~28 days of water temperatures), and exposures x represented by the standardised temperatures experienced by population within L. Within this context, $l_{0}$ is considered the last hour before samples were removed from the stream. The time during which the individuals were transported to the University and exposed to thermal ramping to assess CT_max_ is not included in this analysis but nonetheless was approximately the same for all groups. We used 95% confidence intervals to identify significant regions of the response surface $\beta_{x,l}$. We interpreted confidence itervals consistently >0 (exposure–lag combinations consistently increasing $\bar{{\mathrm{CT}}_{\max}}$) or < 0 (decreasing CT_max_) as being significant. The response surface $\beta_{x,l}$ has been centred to 0, meaning that exposures = 0 have no effect on $\bar{{\mathrm{CT}}_{\max}}$ variation.

All analyses were performed in R (R Core Team, 2022a, 2022b) and R Studio (Rstudio Team, 2020) using the packages dlnm (Gasparrini et al., 2020) and mgcv (Wood, 2017).

## 3 RESULTS

### 3.1 Sampling location water temperatures and *S. ignita/B. rhodani* CT_max_

The eight sampling locations were characterised by different water temperature trends over the study period (Figure 1). We do not formally analyse drivers of these differences, but a summary is included in the Appendix S1: SM3. In general, sites downstream of dams exhibited lower temperature variability, a well‐known impact of damming (Ahmad et al., 2021). The main difference in thermal exposures is driven by this reduced variability (i.e. Glenquey Reservoir and Loch of Lintrathen), with Glenquey Reservoir being the coolest sampling location.

Values of CT_max_ varied among species, sampling locations and sampling times (Figure 3). Overall, (1) *B. rhodani* larvae had a lower $\bar{\;{\mathrm{CT}}_{\max}}$. (29.8°C ± 2.2°C) than *S. ignita* (35.4 ± 3.0°C; *t* = −20.8, *p* = <.0001); (2) for each location, the $\bar{\;{\mathrm{CT}}_{\max}}$ calculated for each sampling event varied across sampling events (Figure 3); and (3) no effect of the factors downstream/upstream and dammed/natural on the population CT_max_ was evident.

### 3.2 DLNMs

A total of 24 DLNMs were fitted using different parameterisations for the cross‐basis object, testing for each combination of linear function and cubic regression splines for exposure and lag dimensions. Table 1 reports parameterisation details for each model and the resulting AIC and deviance explained values (Wood, 2017). The sample size allowed us only sufficient power to implement the cross‐basis object with a maximum of three *df*.

**TABLE 1:** Parameterisation and performance of the implemented DLNMs. Model = reference code for each model as reported in the text; x = exposure variable; $f\left(x\right)$ = parameterisation of exposure dimension; $w\left(l\right)$ = parameterisation of lag dimension; intercept = inclusion (Yes) of exclusion (No) of the intercept on the at $l_{0}$. Lin = linear, cr = cubic regression. Best‐fit models for impacts of absolute temperature (*Zs* vs. ${\mathrm{Tr}}_{\mathrm{Di}}$ transformed) and temperature rate variation (*Atr*) on CT_max_ are highlighted in bold.

Model	x	$f\left(x\right)$	$w\left(l\right)$	Intercept	Model performance	
					AIC	ED%
Zs1	Zs	lin	lin	Yes	93.1	86.9
Zs2		lin	lin	No	91.3	87.8
Zs3		cr, df = 3	cr, df = 3	Yes	**91.0**	**89.0**
Zs4		cr, df = 3	cr, df = 3	No	92.2	88.4
Zs5		cr, df = 3	lin	Yes	93.1	86.9
Zs6		cr, df = 3	lin	No	91.3	87.8
Zs7		lin	cr, df = 3	Yes	91.1	88.9
Zs8		lin	cr, df = 3	No	92.2	88.4
Tr_Di_1	${\mathrm{Tr}}_{\mathrm{Di}}$	lin	lin	Yes	88.5	90.3
Tr_Di_2		lin	lin	No	90.7	88.4
Tr_Di_3		cr, df = 3	cr, df = 3	Yes	**87.1**	**91.3**
Tr_Di_4		cr, df = 3	cr, df = 3	No	88.0	90.8
Tr_Di_5		cr, df = 3	lin	Yes	91.2	87.0
Tr_Di_6		cr, df = 3	lin	No	90.2	87.5
Tr_Di_7		lin	cr, df = 3	Yes	87.3	91.1
Tr_Di_8		lin	cr, df = 3	No	90.7	88.4
At_r_1	${\mathrm{At}}_{r}$	lin	lin	Yes	89.5	89.3
At_r_2		lin	lin	No	92.2	87.4
At_r_3		cr, df = 3	cr, df = 3	Yes	**74.6**	**95**
At_r_4		cr, df = 3	cr, df = 3	No	81.9	89.9
At_r_5		cr, df = 3	lin	Yes	80.9	93
At_r_6		cr, df = 3	lin	No	82.3	91.4
At_r_7		lin	cr, df = 3	Yes	89.5	89.3
At_r_8		lin	cr, df = 3	No	88.7	90.4

All the models based on Zs standardised water temperature had a lower performance (higher AIC and lower deviance explained) compared to equivalent models based on ${\mathrm{Tr}}_{\mathrm{Di}}$ and At_r_ standardisation. Of the ${\mathrm{Tr}}_{\mathrm{Di}}$ models, Tr_Di_3 was the best model for explaining the effect of standardised absolute water temperature on $\bar{\;{\mathrm{CT}}_{\max}}$. The effect of temperature rate of change was best captured by the At_r_3 model.

In all *Tr*_*Di*_ and *At*_*r*_ models, the inclusion of the intercept on the lag dimension improved model performance. Practically, this implies that temperatures close to $l_{0}$ have the potential to modify $\bar{\;{\mathrm{CT}}_{\max}}$. The positive effect for the model performance (AIC) of the inclusion of the intercept is evident for the *At*_*r*_ models, but it had a more limited effect on *Tr*_*Di*_. In addition, all models performed better when exposure and lag space were parameterised with a non‐linear function, indicating non‐linearity of $f\left(x\right)$ and $w\left(l\right)$.

**FIGURE 4:**
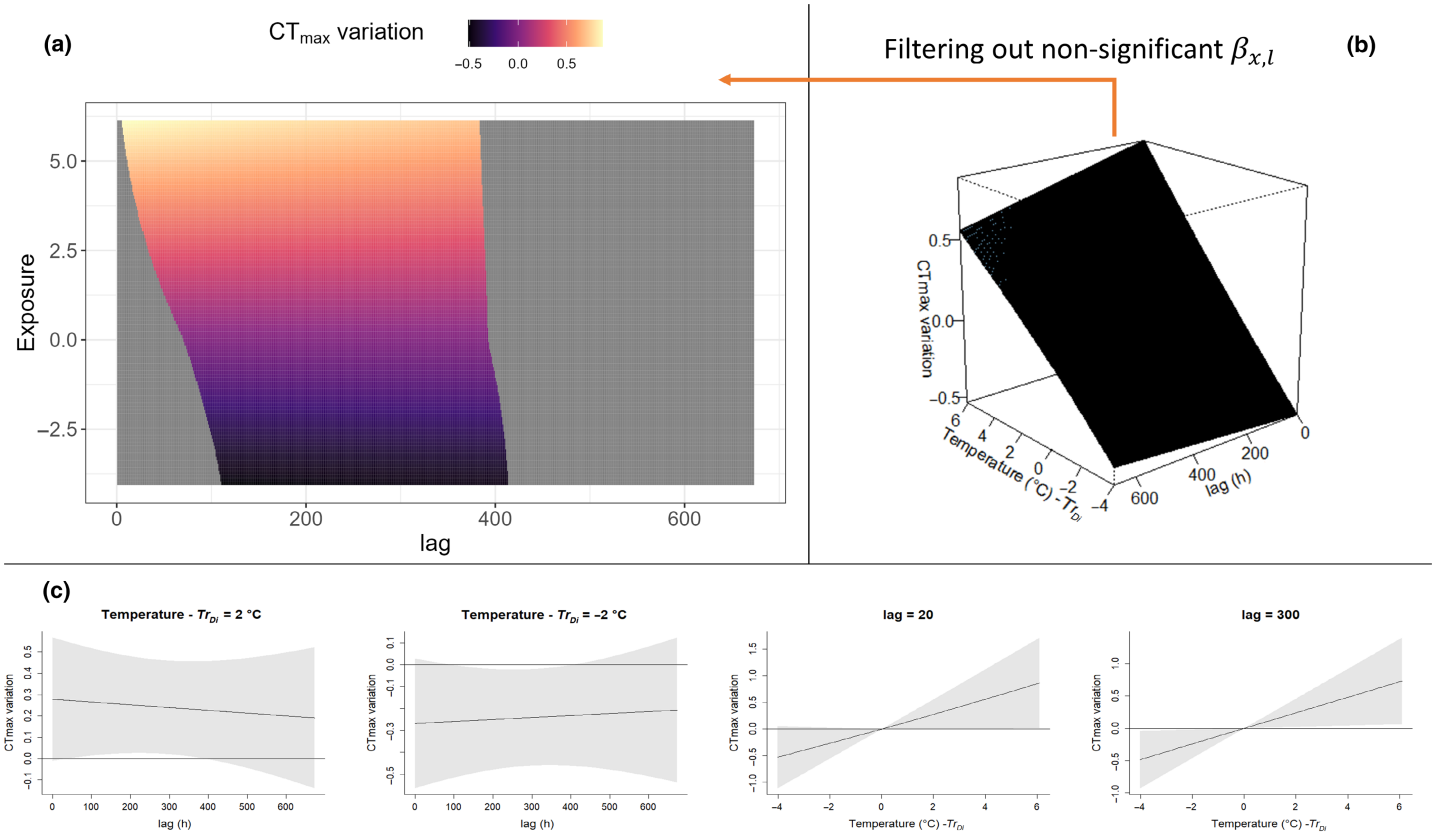
DLNM plot for effects of temperature magnitude, ${\mathrm{Tr}}_{\mathrm{Di}}$ standardised, on $\bar{\;{\mathrm{CT}}_{\max}}$ estimated with ${\mathrm{Tr}}_{\mathrm{Di}}$3 model. The amount of temperature deviation from the modelled trend (${\mathrm{Tr}}_{\mathrm{Di}}$) significantly impacts $\bar{\;{\mathrm{CT}}_{\max}}$ over intermediate timescales, consistent with longer‐term acclimation processes over 1–3 weeks. (a) 2D heatmap of the overall surface, the grey area corresponds to $\beta_{x,l}$ where the 95% CI includes 0. (b) Default overall plot from dlnm package of the ${\mathrm{Tr}}_{\mathrm{Di}}$ – lag–response surface. (c) Default plot offered by the ‘dlnm’ package, slicing the surface on the lag and exposure dimension; by way of example, we sliced at lag = 20, lag = 300, ${\mathrm{Tr}}_{\mathrm{Di}}$= − 2°C and +2°C; shading represents 95% CI.

**FIGURE 5:**
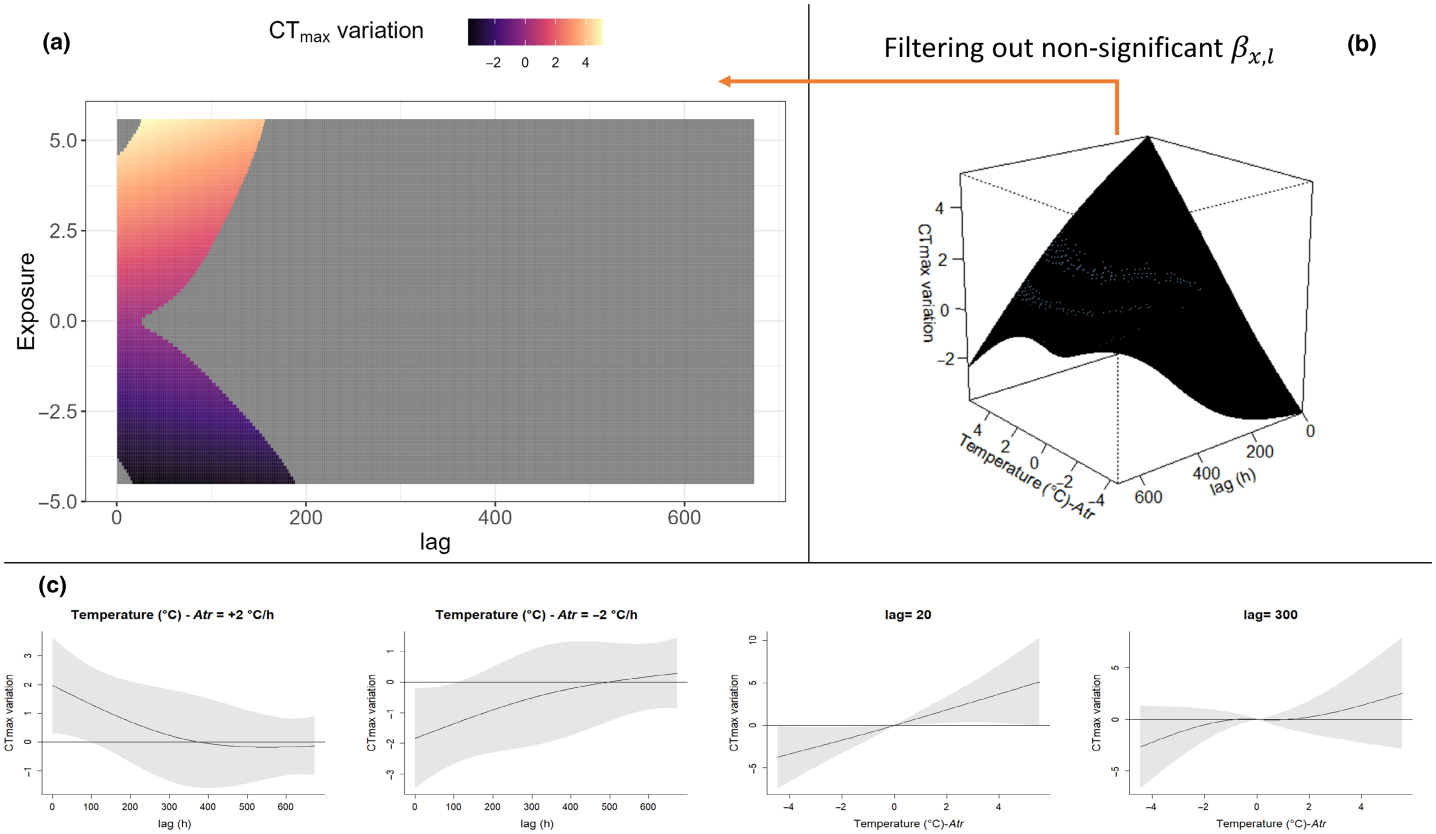
DLNM plot for effect of Temperature Change Rate on $\bar{\;{\mathrm{CT}}_{\max}}$ estimated with ${\mathrm{At}}_{r}$3 model. Rapid and dramatic rates of change in ambient temperature impact $\bar{\;{\mathrm{CT}}_{\max}}$ over shorter timescales than do changes in absolute temperature deviations from the trend (compared to Figure 4). (a) 2D heatmap of the overall surface; the grey area corresponds to $\beta_{x,l}$ where the 95% CI includes 0. (b) Default overall plot from dlnm package of the ${\mathrm{At}}_{r}$ – lag–response surface. (c) default plot offered by the ‘dlnm’ package, slicing the surface on the lag and exposure dimension; by way of example, we sliced at lag = 20, lag = 300, ${\mathrm{At}}_{r}$ = −2°C/h and +2°C/h; shading represents 95% CI.

Following model selection, best‐fit models Tr_Di_3 and At_r_3 were used to predict the exposure–lag–response surfaces. As a comparison, we report the exposure–lag–response surfaces predicted with the *Zs* standardised temperatures. The effects of Zs, TrDi and Atr were estimated over the range of the available data (Figures 4 and 5). The surface can be interpreted as the contribution from a unit increase in exposure x (${\mathrm{Tr}}_{\mathrm{Di}}$ and ${\mathrm{At}}_{r}$ respectively), occurring at time $t-l_{i}$ in the past to a given $\bar{\;{\mathrm{CT}}_{\max}}$ measured at time *t*. ${\mathrm{Tr}}_{\mathrm{Di}}$ had a maximum positive effect on $\bar{\;{\mathrm{CT}}_{\max}}$ of 0.87°C for ${\mathrm{Tr}}_{\mathrm{Di}}$= +6.1°C at l=6 h, and a maximum negative effect of −0.51°C for a ${\mathrm{Tr}}_{\mathrm{Di}}$ = −4.0 at l= 111 h. ${\mathrm{Tr}}_{\mathrm{Di}}$ had no significant immediate effect in the $\bar{\;{\mathrm{CT}}_{\max}}$ at l = 0. Significant $\beta_{x,l}$ occurred roughly from 6 to 400 h (maximum positive ${\mathrm{Tr}}_{\mathrm{Di}}$) or 160 to 411 h (maximum negative ${\mathrm{Tr}}_{\mathrm{Di}}$).

Interestingly, exposure to cooler temperature deviations appears to significantly reduce CT_max_ (potentially representing a form of heat‐tolerance de‐acclimatisation, which has received little empirical attention to date). De‐acclimatisation processes, however, occur on slightly longer timescales than acclimatisation (Figure 4). Also, even very minor temperature deviations from the trend (${\mathrm{Tr}}_{\mathrm{Di}}$ close to 0°C, the centring values for the exposure–lag–response surface) are estimated to have a significant effect on $\bar{\;{\mathrm{CT}}_{\max}}$, although these effects are substantially more minor than those of more extreme temperatures (Figure 5a).

For the best‐fit model examining impacts of rate of temperature change, At_r_3, the effect $\beta_{x,l}$ was estimated to increase $\bar{\;{\mathrm{CT}}_{\max}}$ for positive ${\mathrm{At}}_{r}$ values and decrease $\bar{\;{\mathrm{CT}}_{\max}}$ for negative ${\mathrm{At}}_{r}$ over all significant lags. The maximum positive effect on$\bar{\;{\mathrm{CT}}_{\max}}$ was estimated to be +5.1°C for ${\mathrm{At}}_{r}$ = 5.5°C/h at *l* = 25 and a maximum negative effect of −3.8°C for a ${\mathrm{At}}_{r}$ = −4.5°C/h at *l* = 16. Like the ${\mathrm{Tr}}_{\mathrm{Di}}$3 model, minor temperature shifts (close to ${\mathrm{At}}_{r}$ = 0°C/h) were estimated to have a minor effect on $\bar{\;{\mathrm{CT}}_{\max}}$, and these were not significant after l=20 (i.e. small rates of change have no impact on $\bar{\;{\mathrm{CT}}_{\max}}$). In addition, except for extreme positive and negative exposures, ${\mathrm{At}}_{r}$ has an immediate effect on $\bar{\;{\mathrm{CT}}_{\max}}$ variation (significant at $l_{0}$).

In contrast, estimated *Zs* effects on $\bar{\;{\mathrm{CT}}_{\max}}$ were only significant in a small portion between l=180 and l=210. Moreover, the average predicted effects were marginal (<0.02°C of variation in $\bar{\;{\mathrm{CT}}_{\max}}$) compared to ${\mathrm{Tr}}_{\mathrm{Di}}$ and ${\mathrm{At}}_{r}$ effects, indicating that *Z*‐standardisation results in a poor fit to model the changes in temperature (without respect to underlying trend). Figures are reported in Appendix S1.

## 4 DISCUSSION

The inclusion of lagged effects in ecological studies is still quite limited, although it is more frequent in epidemiology. Recent studies have explored lagged relationships between climate and tree growth (e.g. Nothdurft, 2020) and the effect of hydrologic conditions on freshwater macroinvertebrate community structure (Le et al., 2020). We used a range of DLNMs to provide insights into thermal acclimatisation, indexed using the critical thermal maximum ($\bar{\;{\mathrm{CT}}_{\max}}$). Our focal taxa were the larvae of the freshwater ectotherms *Serratella ignita* and *Baetis rhodani*, with changes in their $\bar{\;{\mathrm{CT}}_{\max}}$ modelled as function of the exposure to temperatures in the 4 weeks preceding sample collection, in natural conditions. Different DLNMs were implemented to assess the effects of absolute water temperature and its rate of change over the evaluated acclimatisation period on $\bar{\;{\mathrm{CT}}_{\max}}$. As part of the work, we also assessed the influence of different methods of standardising water temperature on DLNM performance.

The best DLNMs, used to estimate the temperature–lag–response surface of the focal taxa, were characterised by a slight non‐linearity in both exposure and lag dimension. ${\mathrm{Tr}}_{\mathrm{Di}}$, based on water temperature trend‐standardised absolute temperature, estimated that major effects on $\bar{\;{\mathrm{CT}}_{\max}}$ relate to extreme temperatures (i.e. thermal events that are substantially lower or higher than the trend temperature). It has been highlighted in previous work that organismal responses are commonly triggered by deviations of the environment from the current level of acclimatisation, rather than by the environment's absolute state (Dowd & Denny, 2020). Our work also supports this hypothesis, as *z*‐scaled temperatures performed worse than trend‐standardised temperature predictors. Following *z*‐scaling, the midpoint average of the time period is subject to bias and depends arbitrarily on the time period considered (i.e. potentially capturing an absolute environmental state). *Z*‐scaling may perform better when longer time periods are considered (e.g. multiannual cycles), as these are less likely to be impacted by sampling window. Nonetheless, for time‐series data exhibiting an underlying directional trend, the trend distance (${\mathrm{Tr}}_{\mathrm{Di}}$) is a potentially useful method applicable to any time‐continuous exposures (e.g. temperature, flow, radiation, exposure to chemicals, etc.) that can improve models. Overall, the *At*_*r*_‐based model has the best performance (Table 1); nonetheless, according to Figures 4 and 5. *Tr*_*Di*_ and *At*_*r*_ models seem to capture different processes, happening with different delay times and magnitudes. The model based on *At*_*r*_ estimated that very fast temperature change rates (both positive or negative) have an immediate effect on $\bar{\;{\mathrm{CT}}_{\max}}$ (i.e. the effect significant at and thus cumulated up to the last hour before sampling), while smaller change rates seem not to impact $\bar{\;{\mathrm{CT}}_{\max}}$ significantly at any time period. Additionally, the magnitude of the effect of temperature change rate (*At*_*r*_) is substantially higher (altering $\bar{\;{\mathrm{CT}}_{\max}}$ by as much as between +5.3 and − 4.1°C), compared to the estimated effect of absolute temperature deviations (${\mathrm{Tr}}_{\mathrm{Di}}$3; Figures 4, 5). This suggests that rapid hardening responses are both more dramatic and more transient than the ${\mathrm{Tr}}_{\mathrm{Di}}$‐induced effect. Rather, ${\mathrm{Tr}}_{\mathrm{Di}}$ effects operate with a delay, different in their positive (acclimatisation) and negative (de‐acclimatisation) effects, and being slower to impose their effect (Figure 4), confirming comparable laboratory‐based studies on mussels (Moyen, Somero, & Denny, 2020). To our knowledge, no previous work has assessed acclimatisation mechanisms to thermal exposures from field data such as used here. Usually the relationship between CT_max_ and different thermal regimes is built by summarising temperature profiles through conventional metrics (Dallas & Rivers‐Moore, 2012), which may therefore differ between populations of the same species exposed to different regimes. More typically, acclimatisation responses (established through CT_max_ variations) are assessed in laboratory experiments, where individuals are exposed to thermal regimes lacking variation or stochasticity prior to CT_max_ testing (but see, for instance, Kingsolver et al., 2016; Salinas et al., 2019; Paaijmans et al., 2013). Thus, a quantitative comparison between our findings (i.e. amount of delay, magnitude of effect, etc.) and the existing literature is not possible. Despite this, our findings align with existing knowledge of thermal acclimatisation mechanisms. As reported by recent review (Bates & Morley, 2020), biochemical and molecular changes associated with longer‐term acclimatisation that allow a stable physiological state and maximisation of individual performance in a new thermal environment operate at the timescale of days to weeks, while resistance mechanisms associated with rapid hardening (i.e. the production of heat‐shock protein/switch to anaerobic metabolisms that preserve cells integrity and come with a high energetic cost) operate at scales of seconds to a few days (e.g. Bowler, 2005; Moyen, Crane, et al., 2020; Moyen, Somero, & Denny, 2020; Sørensen et al., 2019) and are able to increase CT_max_ by several°C, as found with our DLNMs. Importantly, rapid hardening mechanisms have been interpreted as temporary and bridging the period before acclimatisation takes place (Bates & Morley, 2020). Interestingly, ${\mathrm{Tr}}_{\mathrm{Di}}$3 and At_r_3 seem to fit with these two mechanisms, previously determined largely from laboratory study, in terms of both timescale and magnitude of impact. In fact, At_r_3 effects operate on CT_max_ in the time period where Tr_Di_3 effects are still not significant (Figures 4, 5), following the bridging mechanism cited. This congruence suggests that the use of DLNMs in the wild corroborates more detailed physiological examination in the laboratory while adding additional specificity and nuance in understanding ecologically salient cues and lag periods for these alternative acclimatisation processes.

Our models suggest that extreme values of *Tr*_*Di*_ and *At*_*r*_; affect acclimatisation and de‐acclimatisation processes. Evidence shows that organisms exposed to higher thermal variability show higher upper thermal limits (e.g. Kern et al., 2015; Kingsolver et al., 2016), in agreement with DLNM effect estimation reported here. Nonetheless, our models improve on previous knowledge by quantifying the specific contribution of each single exposure instance at a different lag. We suggest that the significance and magnitude of exposure and lag attributed by the DLNM should be further verified in experimental conditions, to confirm the effective physiological relevance.

Previous studies on acclimatisation in the wild have shown how a response (e.g. magnitude of CT_max_ variation, depending on salient thermal cues) can vary from species to species (e.g. Dallas, 2016; Dallas & Rivers‐Moore, 2012; Gunderson & Stillman, 2015). However, other studies have found that rather than taxonomic differences shaping acclimatisation abilities, shared environmental drivers are the main explanatory variable (Rohr et al., 2018) – populations of the same species can have different acclimatisation abilities when exposed to different environmental conditions (e.g. Donelson & Munday, 2012). By modelling together *B. rhodani* and *S. ignita* $\bar{\;{\mathrm{CT}}_{\max}}$ responses (and as well different populations), we estimated the ‘averaged effects’, while the inclusion of the species term predictor in the model allowed us to control for the difference in absolute CT_max_ between the two species (Equation 4). To test whether this covariate is sufficient to capture species differences in estimating overall responses, we further estimated the species level response of *S. ignita*, the species that had the largest number of individuals in our sample data. The smaller sample for the species‐level analysis resulted in a higher uncertainty in the estimation of effects (Appendix S1); nonetheless we found a strong agreement in the overall estimated effects between the species – combined analysis in the main text and in the *S. ignita* only ananlysis data. DLNM can therefore be a powerful instrument to explore both similarities and differences in acclimatisation mechanisms between different population of same species or by comparing different species.

We modelled *Tr*_*Di*_ and *At*_*r*_ separately, even if we recognise that the two mechanisms can operate simultaneously. The main practical reason for the separation of *Tr*_*Di*_ and *At*_*r*_ was that the number of observations in our dataset did not provide enough degrees of freedom to parameterise multiple cross‐basis objects within the same model, thus addressing contemporary the effect of different exposures (i.e. *Tr*_*Di*_ and *At*_*r*_). We strongly suggest that future DLNM implementation should test larger data sets to allow the development of models able to include multiple cross‐basis objects and provide additional opportunities to verify some of the findings of the present work. For instance, if the focus is understanding the specific cues and timeline of initiating acclimation to acute or short‐term thermal fluctuations, the DLNM should be implemented with the target response (e.g. CT_max_) values determined from various exposure conditions (such as the present study). On the other hand, if the focus is on understanding longer‐term fitness or population responses, those responses (e.g. survival and demographic rates) should be frequently evaluated within the same population(s) over a longer period of time (i.e. increasing the number of available lags, and including multiple life stages or generations). In addition, carefully planned lab experiments (e.g. comparing populations reaching the same absolute temperatures but with different rates of change) could help to isolate the interacting mechanisms trigged by absolute temperature (*Tr*_*Di*_) and temperature rate of change (*AT*_*r*_).

Our sites were chosen to be situated upstream and downstream from dams and natural lakes, which exhibit different variability in water temperature (Ward & Stanford, 1983; Ahmad et al., 2021; Redana et al., 2022; Heggenes et al., 2021; Appendix S1: SM3). We found that different magnitudes and rates of change in environmental temperatures had significant impacts on CT_max_ values, although our study lacked power to detect impacts of damming per se. In general, ectotherm assemblages in low‐variability thermal environments (e.g. tropical or polar areas) are expected to have a limited acclimatisation capacity (Deutsch et al., 2008; Morley et al., 2016; Peck et al., 2014), even if in practice little evidence of this has been found (Gunderson & Stillman, 2015). Consequently, the acclimatisation capacity of ectotherms should be further studied in a comparative and cross‐biome context, to understand if the functional response shape (i.e. lags and intensity) of CT_max_ acclimatisation differs between populations or species evolved in more versus less variable environments, and across natural and anthropogenically impacted habitats and regions; such work will allow us to better characterise regional variability and assess whether acclimatisation maybe help species deal with climate change.

Special attention should be paid to exposure representativeness. It is common practice to assume that spatially discrete exposure measurements (i.e. water temperature loggers) are representative of the exposure experienced by individuals. We acknowledge that this may be a limitation in our study, since movement of larvae, via drifting and crawling (Irvine & Henriques, 1984; Winterbottom et al., 1997), may mean that some of the individuals used for assessment of CT_max_ did not experience the thermal regimes represented by the temperature data obtained in the area from which they were collected. Recent studies of spatial heterogeneity of water temperature demonstrate that particular features (shaded areas, channel morphology, and water mixing in confluence zones) can alter absolute water temperature and its temporal regime by several degrees (e.g. Kuhn et al., 2021; Tonolla et al., 2010). We collected samples to avoid the confounding influence of such features, but it remains possible that individuals moved around. Direct attachment of thermal data loggers to individuals can help resolve this problem for larger species (e.g. Kraus et al., 2022).

Our study shows that DLNMs have great utility for understanding biological responses to environmental change, especially in the current era characterised by dramatic and rapid thermal changes in many different environments. These models are a powerful statistical tool to explore the ecological effects of environmental extremes, especially because they allow consideration of temporal dynamics and lagged responses.

## AUTHOR CONTRIBUTIONS

**Matteo Redana:** Conceptualization (equal); data curation (equal); formal analysis (lead); methodology (lead); software (equal); validation (equal); writing – original draft (equal); writing – review and editing (equal). **Chris Gibbins:** Conceptualization (equal); data curation (equal); formal analysis (supporting); funding acquisition (supporting); methodology (supporting); project administration (supporting); resources (equal); software (equal); supervision (equal); validation (equal); visualization (supporting); writing – original draft (equal); writing – review and editing (equal). **Lesley T. Lancaster:** Conceptualization (equal); data curation (equal); formal analysis (supporting); funding acquisition (lead); investigation (lead); methodology (supporting); project administration (equal); resources (lead); software (equal); supervision (equal); validation (equal); visualization (supporting); writing – original draft (equal); writing – review and editing (equal).

## CONFLICT OF INTEREST STATEMENT

None to declare.

## Supporting information


Appendix S1.

## Data Availability

Original Data are available at https://doi.org/10.5061/dryad.4mw6m90gh, Associated code and step‐by‐step tutorials can be found at: https://github.com/monviso/Determining‐Critical‐Periods‐for‐Thermal‐Accliamtion‐Using‐a‐DLNM‐approach.
