# Supplementary material for: Determining critical periods for thermal acclimatisation using a distributed lag non‐linear modelling approach
Source: Ecol Evol. 2024 May 31;14(6):e11451. doi: 10.1002/ece3.11451 (PMC11140238; doi:10.1002/ece3.11451)
Supplement: Supplementary file 1 — Appendix S1. [file ECE3-14-e11451-s001.docx]

**SUPPORTING MATERIAL**

1. **PREDICTION OF WATER TEMPERATURE**
   1. **Material and Method**

Hourly water temperature measurements for each sampling location are not available for the whole time series considered in our study (28 days preceding each sampling). This was due to loggers’ failure, washout and data loss. In Table SM1 we report for each CT_max_ sampling event the number of days which water temperature needed to be predicted due to data loss, to reconstruct the full 28 thermal history days prior of each sample considered in the paper. In all cases, the missing days of temperature recording correspond to earliest period of the thermal histories (i.e., the furthest exposures from the samplings).

Table SM1: sampling dates and number of days of missing water temperature observation for each sampling date and location to have the full 28-days long thermal history. The last column reports the last temperature measured by the logger before the sampling took place.

| Site (upstream and downstream) | Sampling date | location | Missing days | Last temperature experienced (°C) |
| --- | --- | --- | --- | --- |
| Loch Morie | 14/07 | upstream | 0 | 13.9 |
|  | 28/07 | upstream | 1 | 13.7 |
|  |  | downstream | 1 | 14.4 |
| Glenquey Reservoir 2016 | 19/07 | upstream | 0 | 9.8 |
|  |  | downstream | 0 | 13.2 |
|  | 03/08 | upstream | 0 | 8.8 |
|  |  | downstream | 0 | 13.0 |
| Glenquey Reservoir 2017 | 07/07 | upstream | 27 | 10.0 |
|  |  | downstream | 5 | 13.5 |
|  | 12/07 | upstream | 22 | 9.4 |
|  |  | downstream | 0 | 12.6 |
|  | 20/07 | upstream | 14 | 10.1 |
|  |  | downstream | 0 | 13.2 |
| Loch of Lintrathen | 04/07 | upstream | 8 | 11.3 |
|  |  | downstream | 23 | 15.0 |
|  | 11/07 | upstream | 1 | 10.5 |
|  |  | downstream | 16 | 15.9 |
|  | 18/07 | upstream | 0 | 11.5 |
|  |  | downstream | 9 | 16.6 |
| Loch Rescobie | 05/07 | upstream | 22 | 13.2 |
|  |  | downstream | 0 | 16.2 |
|  | 10/07 | upstream | 17 | 13.7 |
|  |  | downstream | 0 | 17.3 |
|  | 19/07 | upstream | 8 | 14.4 |
|  |  | downstream | 0 | 17.9 |

We estimated the missing measurements through the implementation of a set of Generalized Additive Models with different structures (see below) using as predictors of water temperature: (I) air temperature (*Air*), (II) hour of the day (*hour*) and (III) day of the year (*day*). As source of air temperature, we used the closest Met Office meteorological station with hourly air temperature data available through the MIDAS database for the year of interest [1].

The models were all trained on a Training dataset (corresponding to the 75% of the water temperature available period) and validated on a Validation dataset (corresponding with the remaining 25% of the data). Giving that the water temperature data to be estimated were in large part preceding the CT_max_ measurements, the Validation datasets correspond to the earliest 25% of the directly-measured temperature data. The Training datasets were used to implement 7 GAMs for each sampling location (i.e., separately for downstream and upstream of each site). Each GAM was then used to predict the hourly water temperature values of the Validation datasets. For each prediction we computed the Absolute Errors (AE) and consequently the average Mean Absolute Error (mean MAE), the AE standard deviation (AE sd) and the correlation coefficient (r). Models ranked differently in their perfornace according to each of these metrics metrics (i.e., minimum MAE, minimum AEsd and max r). Thus, to select the best model overall, we gave a ranking score from 1 to 10 (i.e., 1 the best model, 10 the worst) to each GAM, for each error metric. The model from which we obtained the lowest sum of the three ranking scores is selected as the best model. In Table SM2 we report for each site the dates with available water temperature data, the corresponding dates of the model training and validation data, and the dates predicted with the selected GAM.

Table SM2: For each sampling locations we report the period of available water temperature data measured in field with HOBO loggers (measured period), the corresponding dates of the training and validation period (Model training period and Model validation period), the period modelled with GAM (Predicted period), the Met Office stations used as air temperature source, and the distance from the sampling location (lake or reservoir centre as reference).

| Site (year) | Measured period  (n° day) | Model training period  (n° day) | Model validation period  (n° day) | Predicted period  (n° day) | Met Office station (MIDAS code) | Distance from sampling site (km) |
| --- | --- | --- | --- | --- | --- | --- |
| Loch Morie  (2016) | 10/06  -  27/07  (48) | 22/06  -  27/07  (36) | 10/06  -  21/06  (12) | 28/07  (1) | Tulloch Bridge (00105) | 97 |
| Glenquey Reservoir  (2017) | 07/07  -  20/07  (14) | 11/07  -  20/07  (10) | 07/07  -  10/07  (4) | 10/06 –  06/07  (27) | Strathallan Airfield (00212) | 15 |
| Loch Lintrathen (2017) | 30/06  -  18/07  (19) | 05/07  -  18/07  (14) | 30/07  -  04/07  (5) | 07/06  -  29/06  (23) | Cairnwell (00145) | 26 |
| Loch Rescobie (2017) | 30/06  –  19/07  (20) | 05/07  -  19/07  (15) | 30/06  -  04/07  (5) | 08/06  -  29/06  (22) | Cairnwell (00145) | 45 |

The selected GAMs were then re-implemented using the whole available time series to increase predictive power and then checked for the validity of the a-priori statistical assumption (error distribution and correlation) through the gam.check() function. Missing water temperature data were then predicted using the selected GAMs. In Figure 1 are schematized all the steps followed to estimate the missing water temperature values.


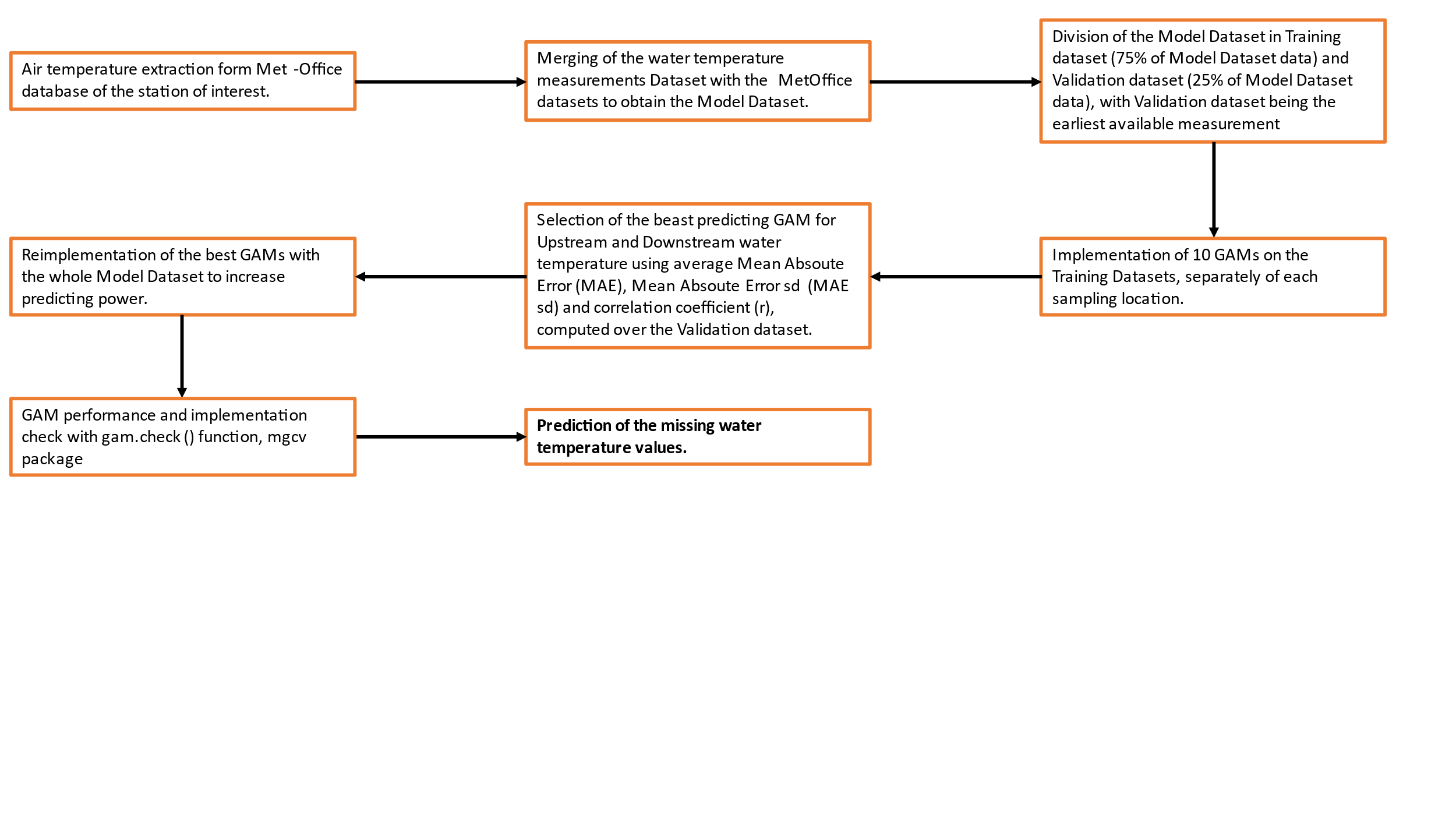


Figure SM1: schematization of the procedure followed to estimate missing water temperature from sampling locations.

Here following we report the equation of the 7 GAMs model implemented for each sampling location. In Table SM3 are reported the specific implementation of each function and the relative number of knots selected. The number of knots for each function was selected following the gam.check() function output (see. [2]) and the available degrees of freedom of each Training dataset. We assumed $\varepsilon_{i}$ is an independent $N\left( 0,\sigma^{2} \right)$ random variable.

1. ${Water}_{temp}=f_{1}\left( Air \right)+\varepsilon_{i}$
2. ${Water}_{temp}=f_{1}\left( Air \right)+f_{2}\left( hour \right)+\varepsilon_{i}$
3. ${Water}_{temp}=f_{3}(Air,hour,day)+\varepsilon_{i}$
4. ${Water}_{temp}=f_{4}(Air,hour)$+ $f_{1}\left( Air \right)+f_{2}\left( hour \right)+\varepsilon_{i}$
5. ${Water}_{temp}=f_{1}\left( Air \right)+f_{2}\left( hour \right)+b_{day}+day+\varepsilon_{i}$
6. ${Water}_{temp}=f_{4}\left( Air,hour \right)+b_{day}+day+\varepsilon_{i}$
7. ${Water}_{temp}=f_{4}\left( Air,hour \right)+f_{1}\left( Air \right)+f_{2}\left( hour \right)+b_{day}+day+\varepsilon_{i}$

Table SM3: GAM function implementation specifications

| Predictors function | Basis-function/s | knots |
| --- | --- | --- |
| $f_{1}$- single smooth term | thin plate spline | k=30 |
| $f_{2}$- single smooth term | cyclic cubic spline | k=24 |
| $f_{3}$ -tensor product | $Air:$ thin plate spline  $hour:$ cyclic cubic spline $day:$ thin plate spline | $Air:$ k=8  $hour:$ k=5  $day:$ k=5 |
| $f_{4}$ -tensor product | $Air:$ thin plate spline  $hour:$ cyclic cubic spline | $Air:$ k=10  $hour:$ k=8 |
| $b_{day}$- day of the year as random effect |  |  |
| $day$- fixed effect for day of the year |  |  |

- 1. **Results**

In Table SM4 are reported the best GAMs foe each sampling location and relative MAE, MAE sd and r. All the models respect the assumption on $\varepsilon_{i}$.

Table SM4: Results for best GAM for each sampling location, reporting Mean Absolute Error (MAE) and its standard deviation (MAE sd), correlation coefficient between validation data and predicted data (r)

| River | year | location | Model | MAE | MAE sd | r |
| --- | --- | --- | --- | --- | --- | --- |
| Morie | 2016 | downstream | 5 | 0.1 | 0.71 | 0.46 |
| Morie | 2016 | upstream | 5 | 0.7 | 1.25 | 0.71 |
| Glenquey | 2016 | downstream | 5 | -0.2 | 0.59 | 0.66 |
| Glenquey | 2016 | upstream | 7 | -0.3 | 0.59 | 0.90 |
| Glenquey | 2017 | downstream | 2 | 0.2 | 0.53 | 0.78 |
| Glenquey | 2017 | upstream | 4 | 0.0 | 0.68 | 0.81 |
| Rescobie | 2017 | downstream | 7 | 1.2 | 0.60 | 0.82 |
| Rescobie | 2017 | upstream | 6 | 0.3 | 0.56 | 0.63 |
| Lintrathen | 2017 | downstream | 6 | -0.3 | 0.24 | 0.65 |
| Lintrathen | 2017 | upstream | 2 | 0.1 | 0.72 | 0.92 |

Here following are reported for each sampling location the timeseries of water temperature values divided in Training dataset and Validation dataset along with the comparison with predicted data from best models.


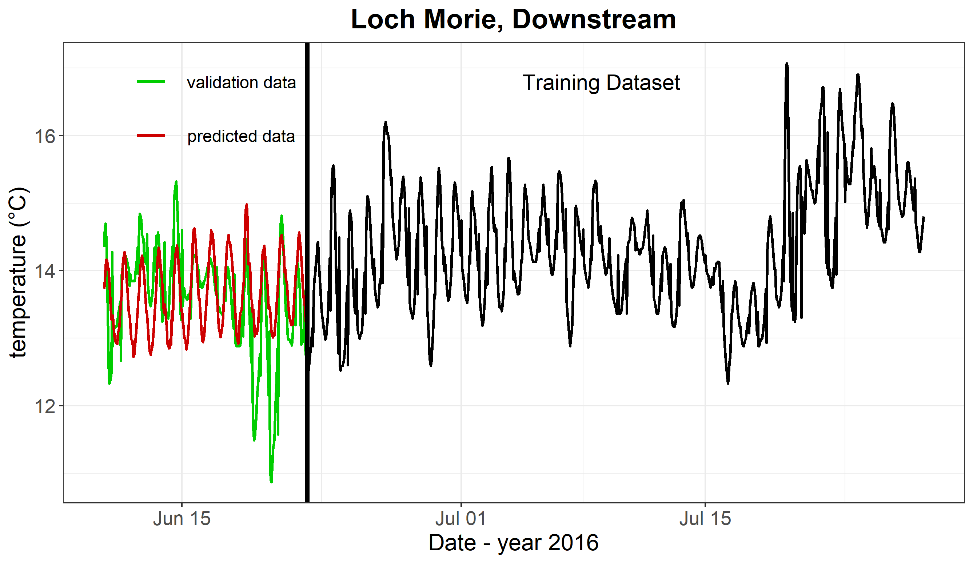


Figure SM2: Loch Morie, year 2016 Downstream.


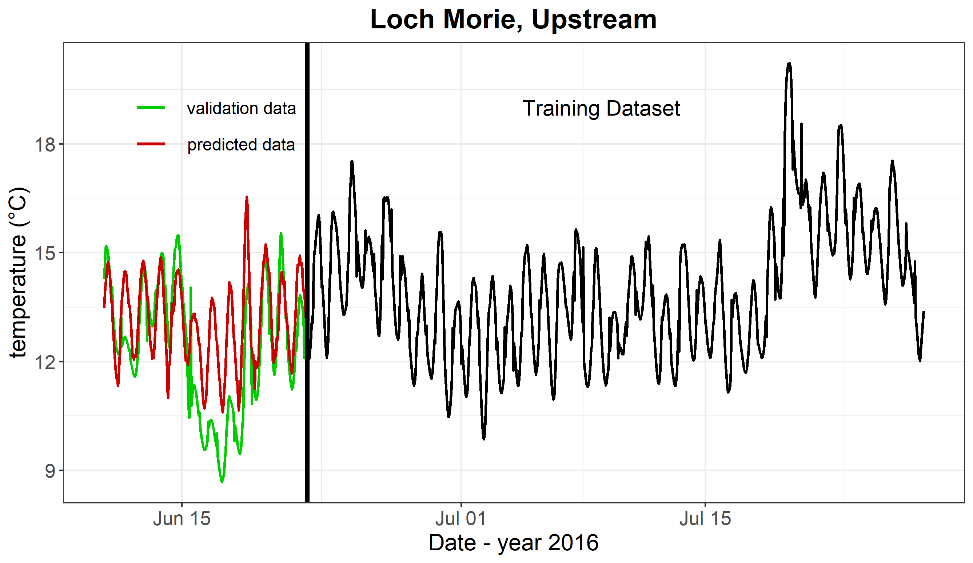


Figure SM3:Loch Morie, year 2016 Upstream.


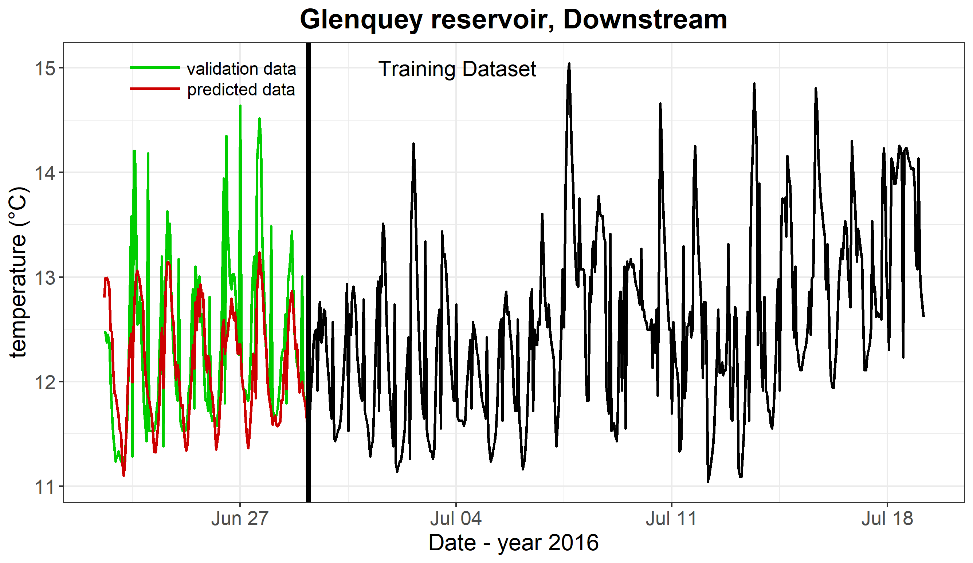


Figure SM4:Glenquey reservoir, year 2016 Downstream.


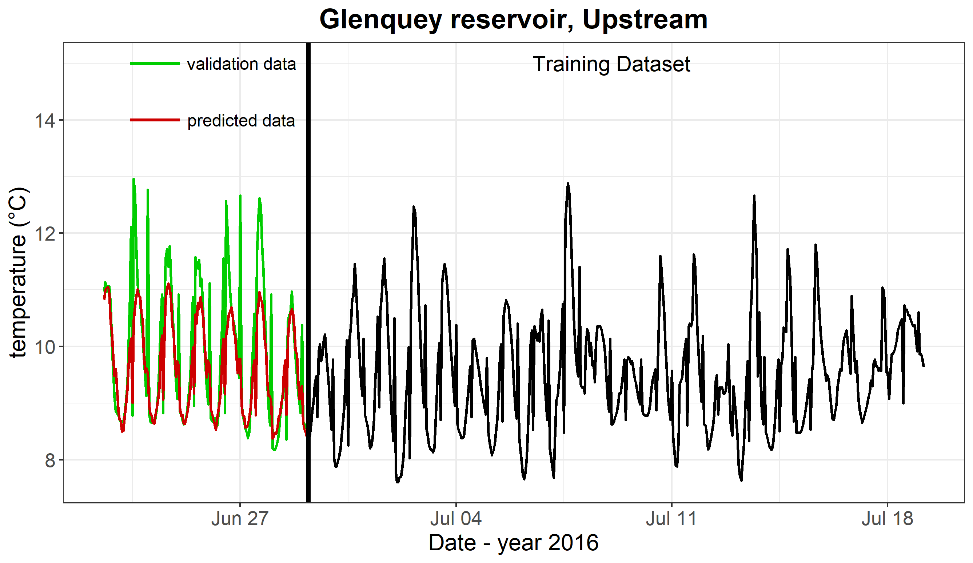


Figure SM5:Glenquey reservoir, year 2016 Upstream.


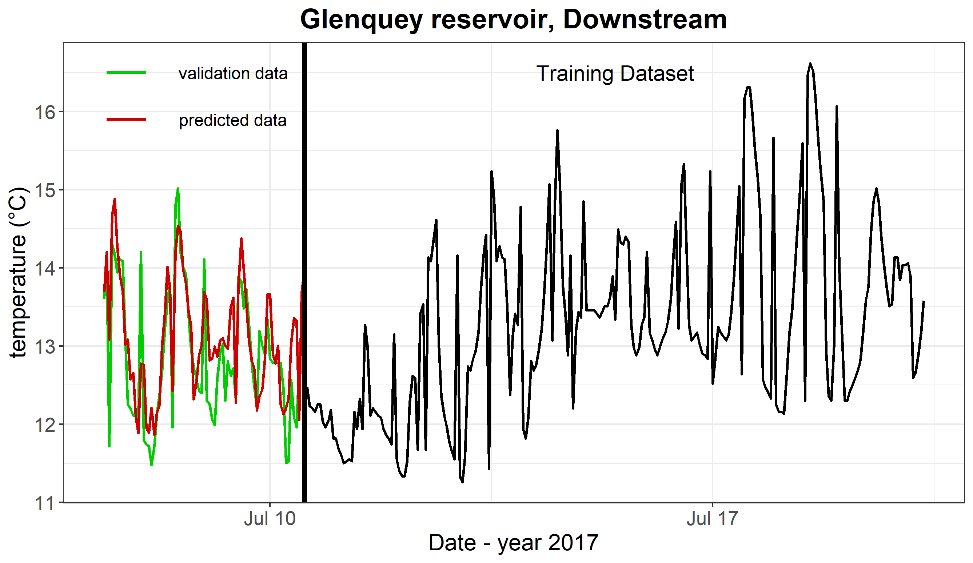


Figure SM6:Glenquey reservoir, year 2017 Downstream.


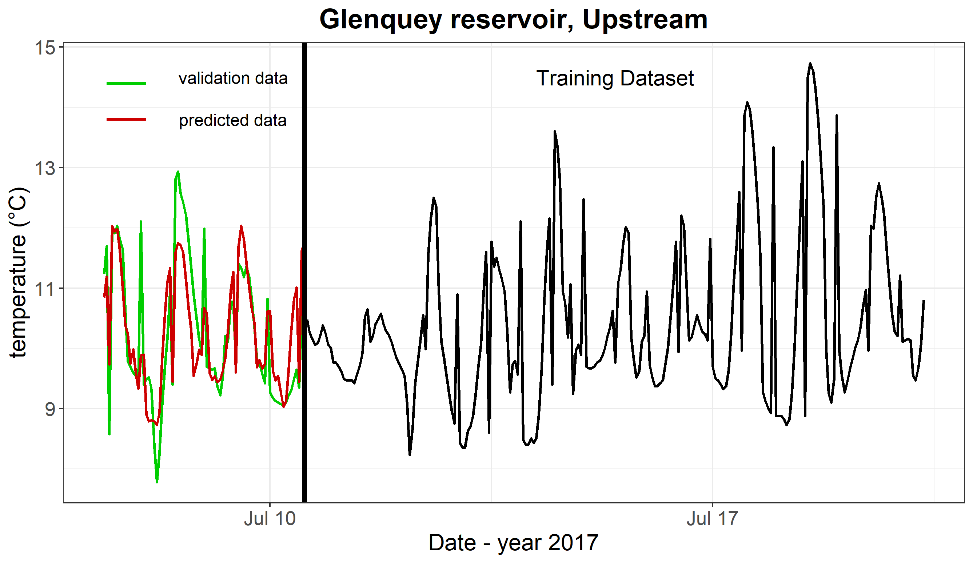


Figure SM7:Glenquey reservoir, year 2017 Upstream.


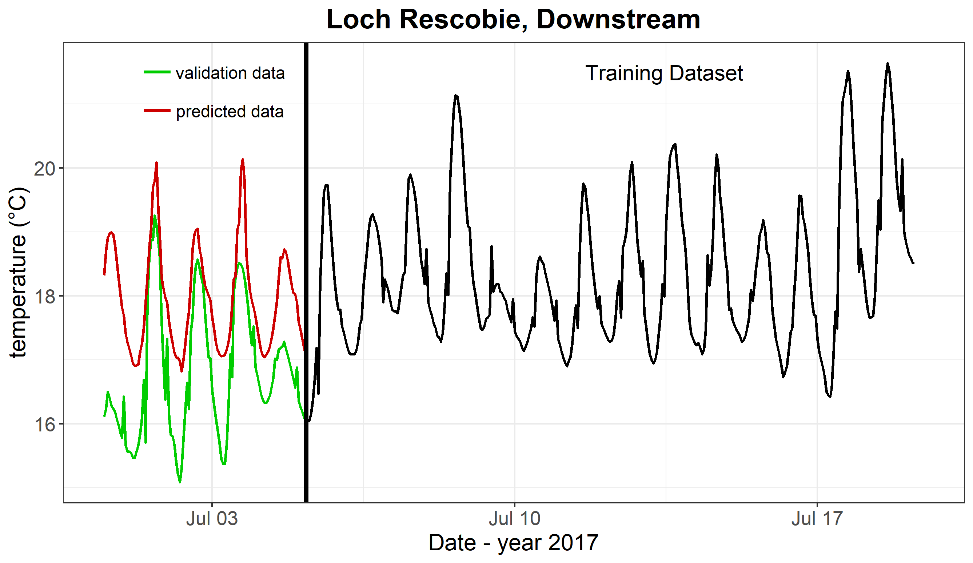


Figure SM8:Loch Rescobie, year 2017 Downstream.


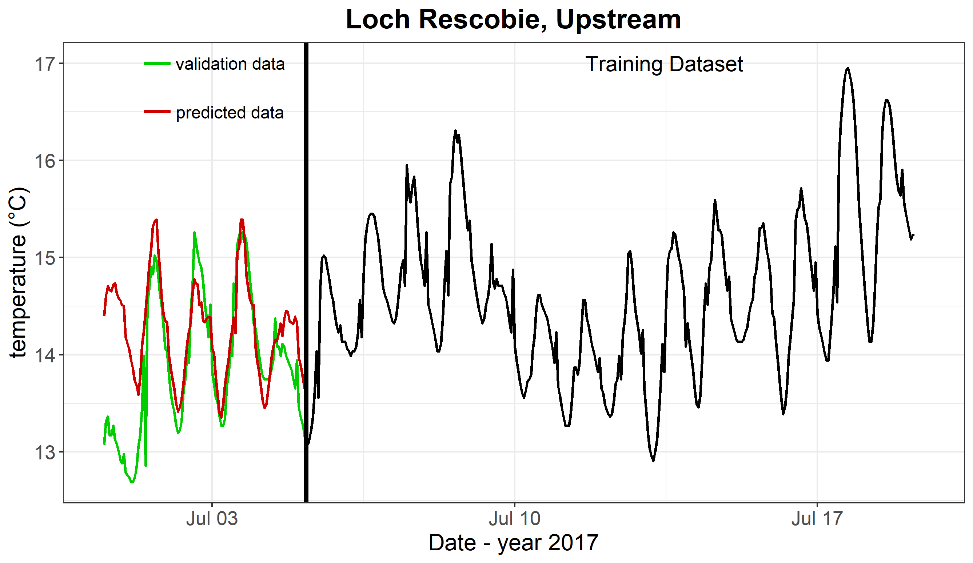


Figure SM9: Loch Rescobie, year 2017 Upstream.


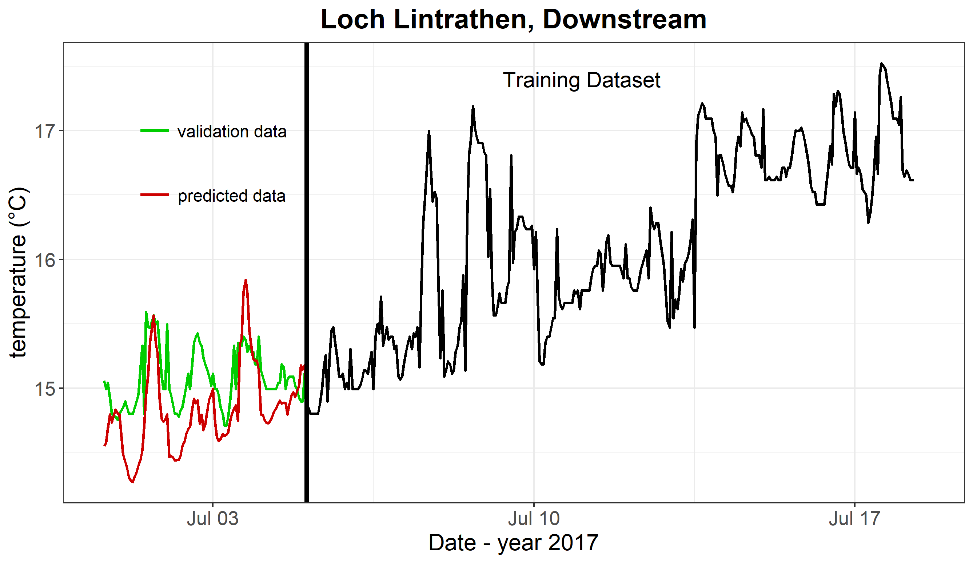


Figure SM10: Loch Lintrathen, year 2017 Downstream.


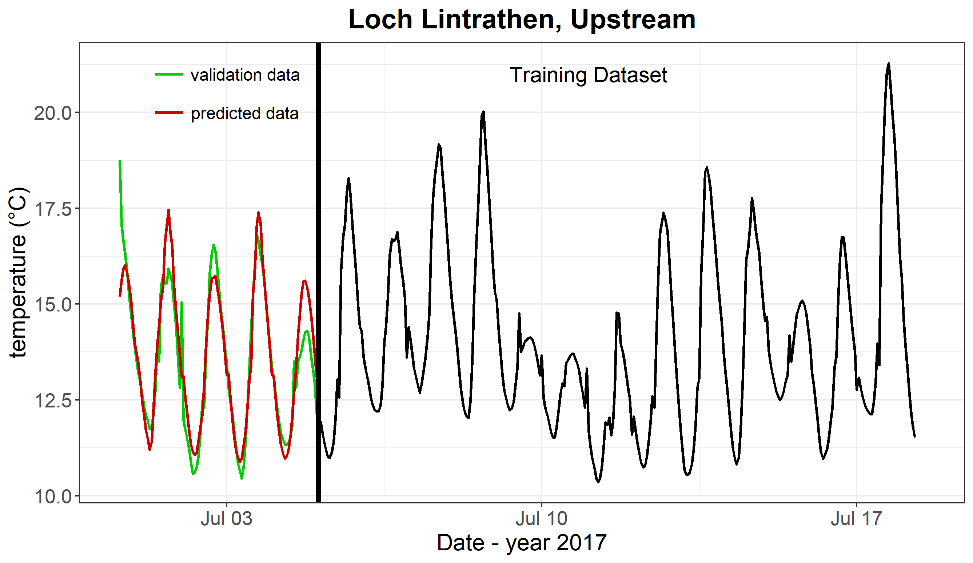


Figure SM11: Loch Lintrathen, year 2017 Upstream.

1. **STANDARDIZED WATER TEMPERAURE VALUES**

We report here for each sampling location 2 main plots: (I) the $Zs$, ${Tr}_{Di}$ standardized absolute temperature and the absolute temperature change rate ${At}_{r}$. See section 2.4 of the main text for details on the computation methods. (II) the comparison in the water temperature standardization as positive or negative values between $Zs$ and ${Tr}_{Di}$. Within the context of our study, positive and negative standardized values have a particular importance. Indeed, positive exposure has been estimated with DLNM ${Tr}_{Di}$3 to increase $\overline{{CT}_{max}}$, and negative exposures to have a decreasing effect on $\overline{{CT}_{max}}$.

LOCH MORIE DOWNSTREAM


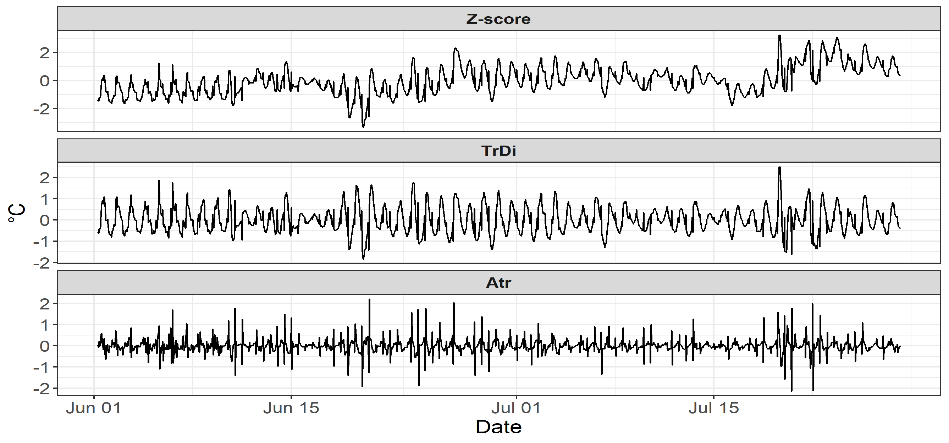

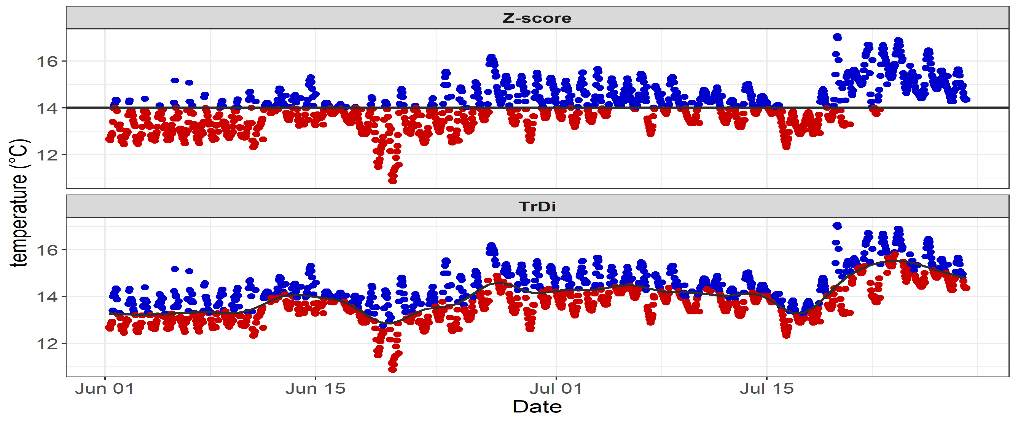


Figure SM12: Loch Morie Downstream standardized temperatures with $Zs$ and ${Tr}_{Di}$ (top 3 row). In the fourth and fifth row are reported water temperature values standardized as positive (**BLUE**) or negative (**RED**) according to $Zs$ and ${Tr}_{Di}$ standardization. The horizontal line in Z-score graph is the mean water temperature during the period, the wiggly line in the ${Tr}_{Di}$ graph is the modelled $Tr$.

LOCH MORIE UPSTREAM


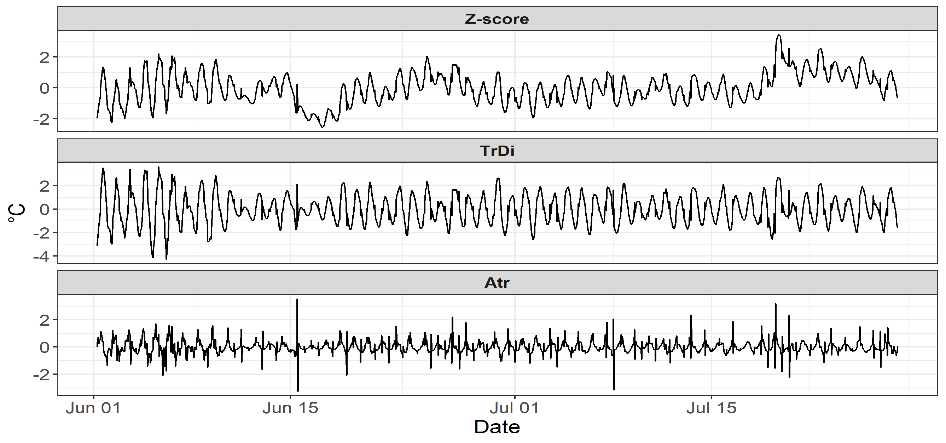

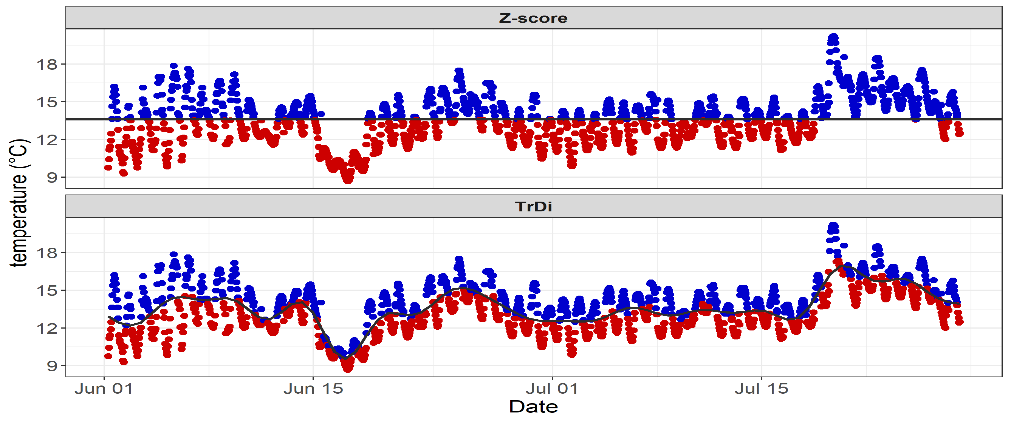


Figure SM13: Loch Morie Upstream standardized temperatures with $Zs$ and ${Tr}_{Di}$ (top 3 row). In the fourth and fifth row are reported water temperature values standardized as positive (**BLUE**) or negative (**RED**) according to $Zs$ and ${Tr}_{Di}$ standardization. The horizontal line in Z-score graph is the mean water temperature during the period, the wiggly line in the ${Tr}_{Di}$ graph is the modelled $Tr$.

GLENQUEY RESERVOIR DOWNSTREAM 2016


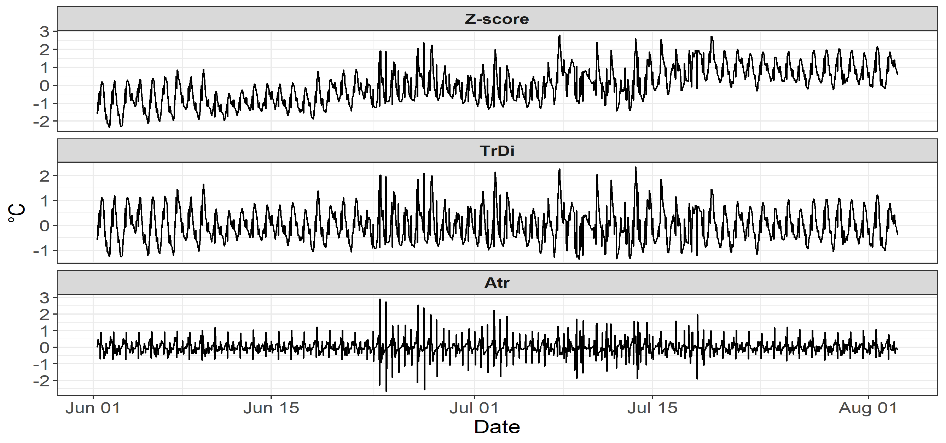

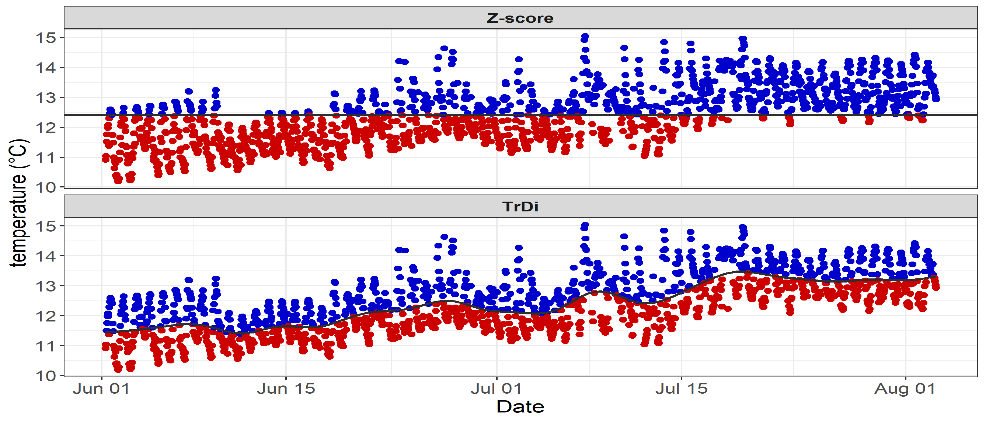


Figure SM14: Glenquey reservoir Downstream, year 2016, standardized temperatures with $Zs$ and ${Tr}_{Di}$ (top 3 row). In the fourth and fifth row are reported water temperature values standardized as positive (**BLUE**) or negative (**RED**) according to $Zs$ and ${Tr}_{Di}$ standardization. The horizontal line in Z-score graph is the mean water temperature during the period, the wiggly line in the ${Tr}_{Di}$ graph is the modelled $Tr$.

GLENQUEY RESERVOIR UPSTREAM 2016


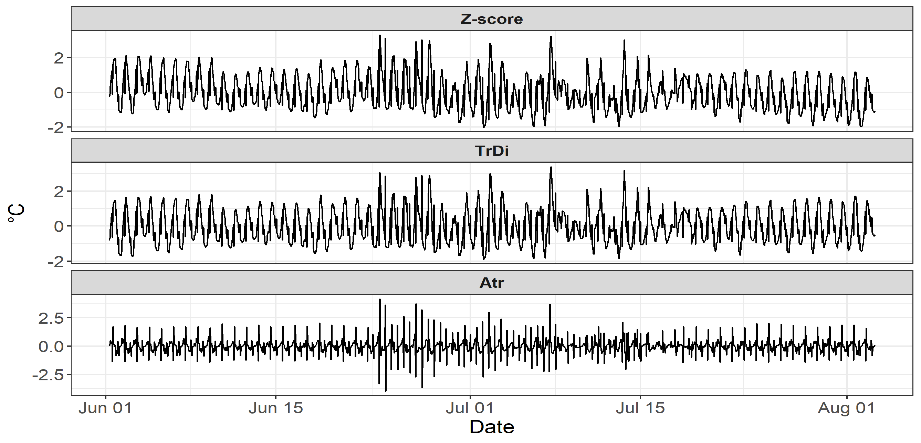

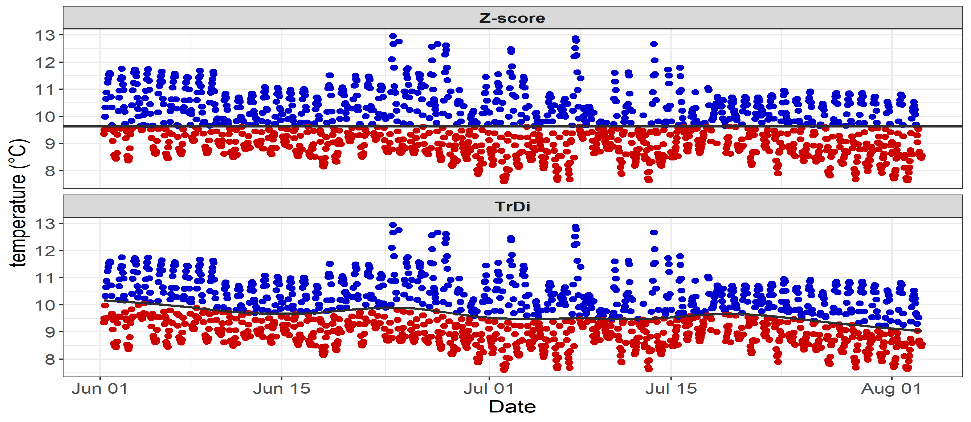


Figure SM15: Glenquey reservoir Upstream, year 2016, standardized temperatures with $Zs$ and ${Tr}_{Di}$ (top 3 row). In the fourth and fifth row are reported water temperature values standardized as positive (**BLUE**) or negative (**RED**) according to $Zs$ and ${Tr}_{Di}$ standardization. The horizontal line in Z-score graph is the mean water temperature during the period, the wiggly line in the ${Tr}_{Di}$ graph is the modelled $Tr$.

GLENQUEY RESERVOIR DOWNSTREAM 2017


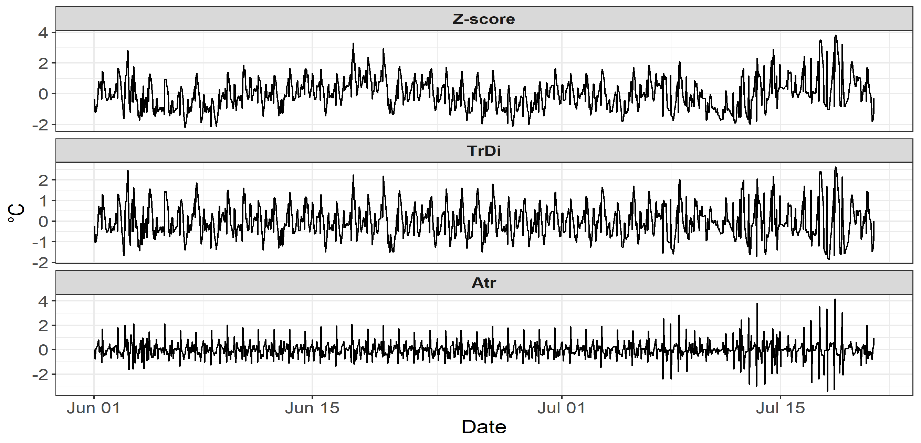

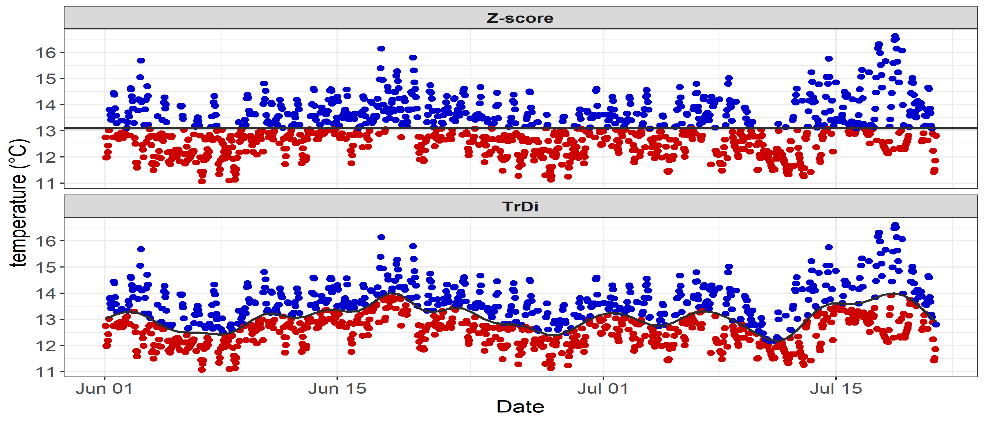


Figure SM16: Glenquey reservoir Downstream, year 2017, standardized temperatures with $Zs$ and ${Tr}_{Di}$ (top 3 row). In the fourth and fifth row are reported water temperature values standardized as positive (**BLUE**) or negative (**RED**) according to $Zs$ and ${Tr}_{Di}$ standardization. The horizontal line in Z-score graph is the mean water temperature during the period, the wiggly line in the ${Tr}_{Di}$ graph is the modelled $Tr$.

GLENQUEY RESERVOIR UPSTREAM 2017


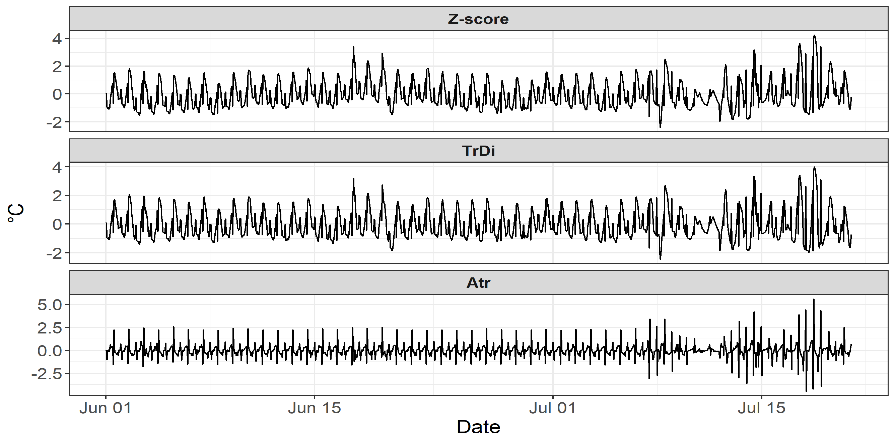

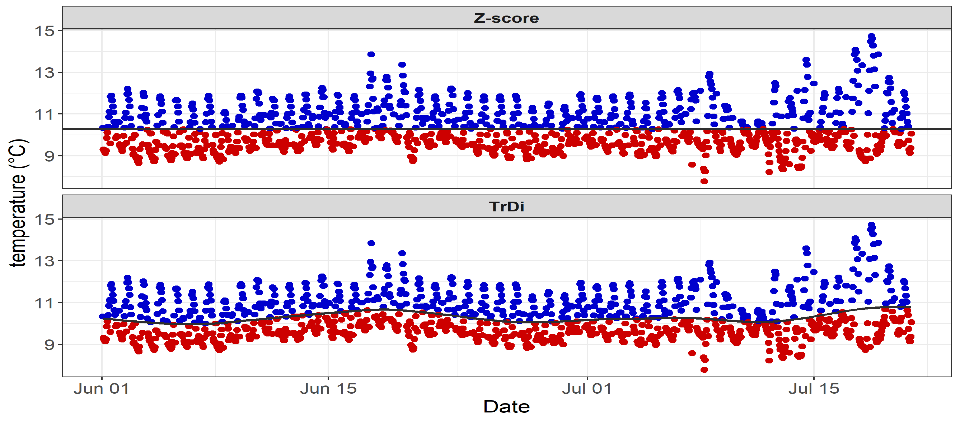


Figure SM17: Glenquey reservoir Upstream, year 2017, standardized temperatures with $Zs$ and ${Tr}_{Di}$ (top 3 row). In the fourth and fifth row are reported water temperature values standardized as positive (**BLUE**) or negative (**RED**) according to $Zs$ and ${Tr}_{Di}$ standardization. The horizontal line in Z-score graph is the mean water temperature during the period, the wiggly line in the ${Tr}_{Di}$ graph is the modelled $Tr$.

LOCH RESCOBIE DOWNSTREAM 2017


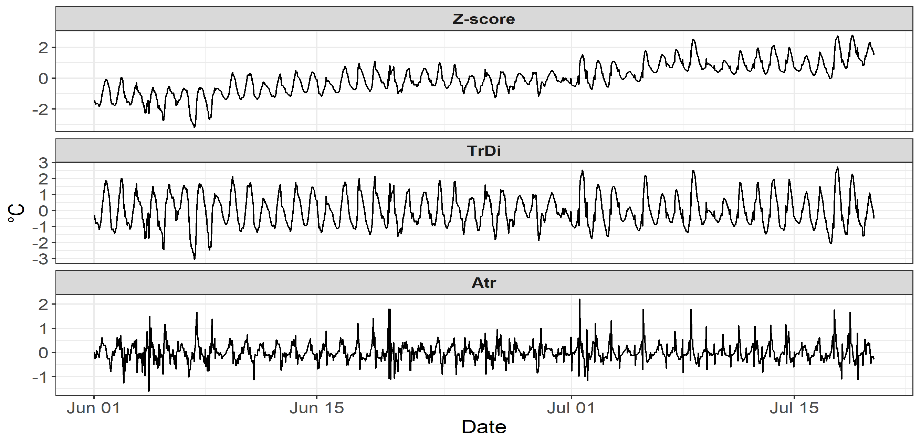

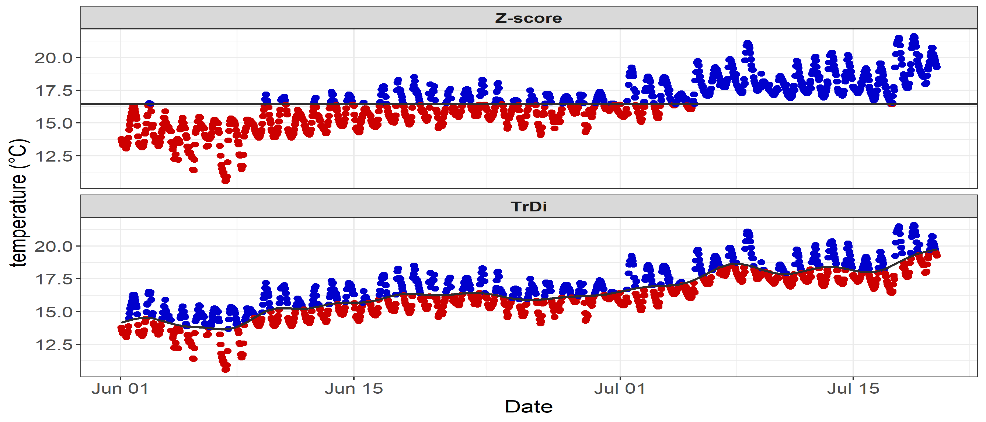


Figure SM18: Loch Rescobie Downstream, year 2017, standardized temperatures with $Zs$ and ${Tr}_{Di}$ (top 3 row). In the fourth and fifth row are reported water temperature values standardized as positive (**BLUE**) or negative (**RED**) according to $Zs$ and ${Tr}_{Di}$ standardization. The horizontal line in Z-score graph is the mean water temperature during the period, the wiggly line in the ${Tr}_{Di}$ graph is the modelled $Tr$.

LOCH RESCOBIE UPSTREAM 2017


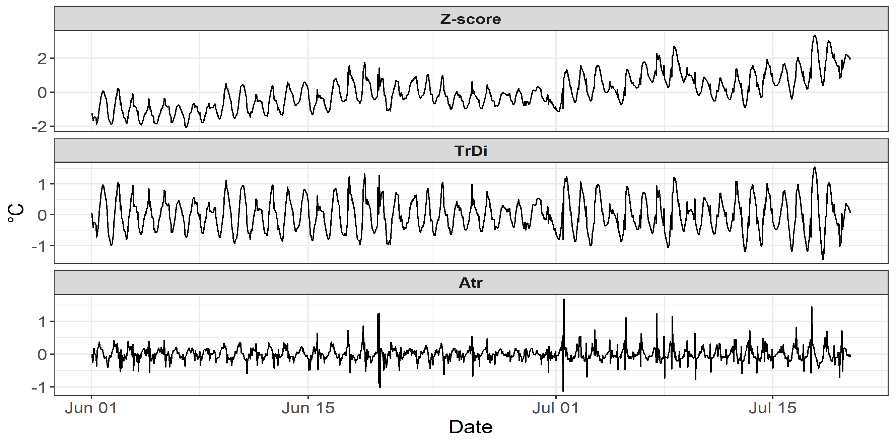

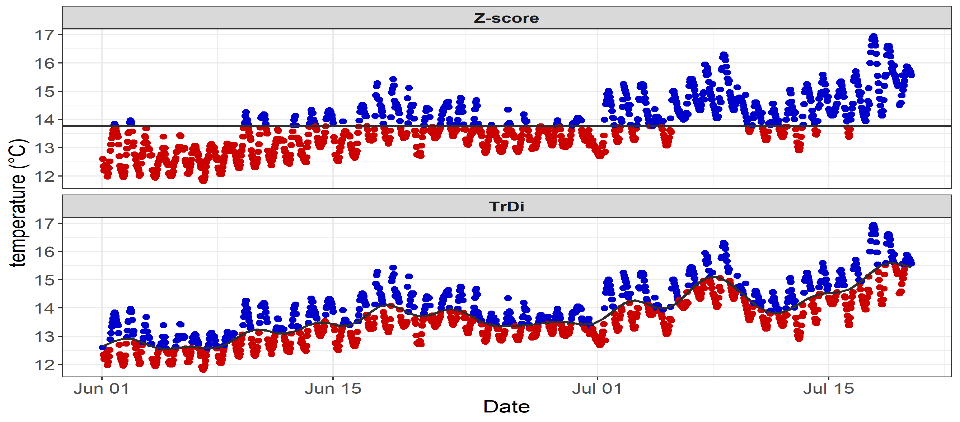


Figure SM19: Loch Rescobie Upstream, year 2017, standardized temperatures with $Zs$ and ${Tr}_{Di}$ (top 3 row). In the fourth and fifth row are reported water temperature values standardized as positive (**BLUE**) or negative (**RED**) according to $Zs$ and ${Tr}_{Di}$ standardization. The horizontal line in Z-score graph is the mean water temperature during the period, the wiggly line in the ${Tr}_{Di}$ graph is the modelled $Tr$.

LOCH LINTRATHEN DOWNSTREAM 2017


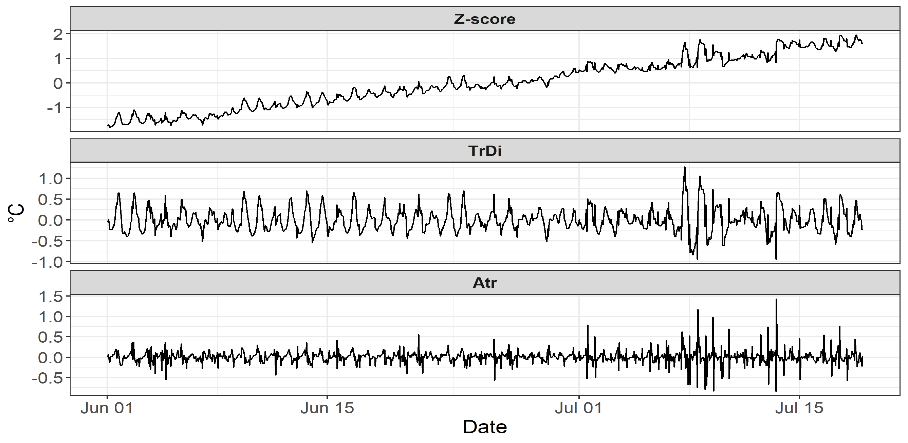

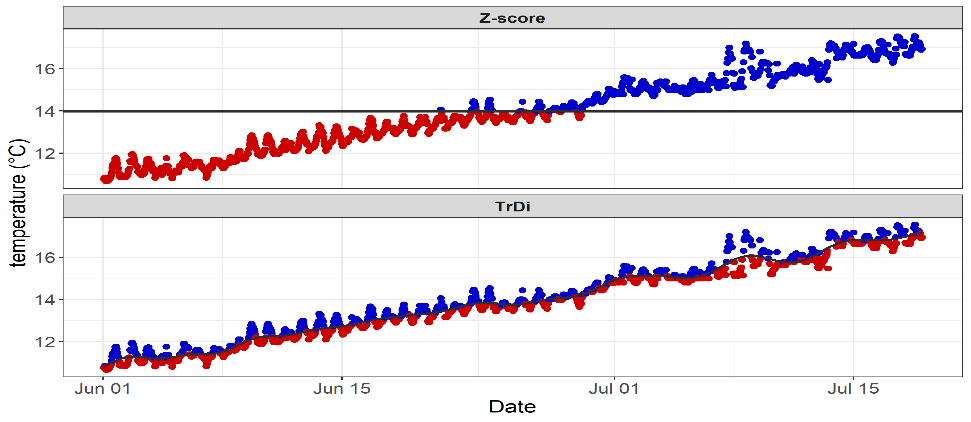


Figure SM20: Loch Lintrathen Downstream, year 2017, standardized temperatures with $Zs$ and ${Tr}_{Di}$ (top 3 row). In the fourth and fifth row are reported water temperature values standardized as positive (**BLUE**) or negative (**RED**) according to $Zs$ and ${Tr}_{Di}$ standardization. The horizontal line in Z-score graph is the mean water temperature during the period, the wiggly line in the ${Tr}_{Di}$ graph is the modelled $Tr$.

LOCH LINTRATHEN UPSTREAM 2017


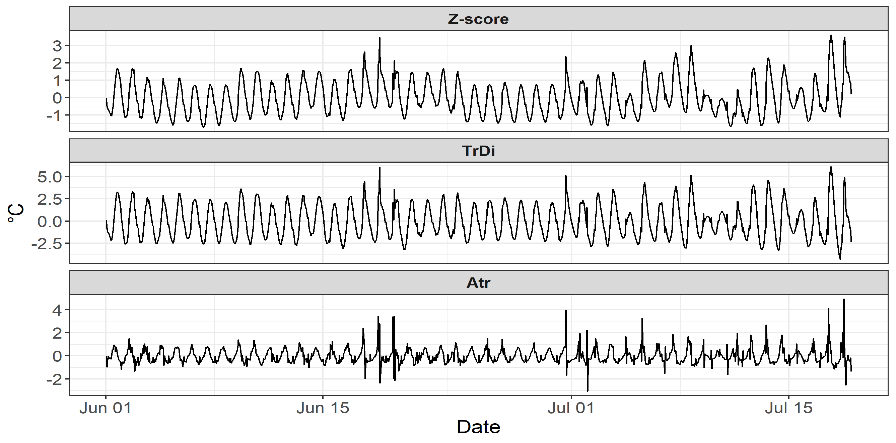

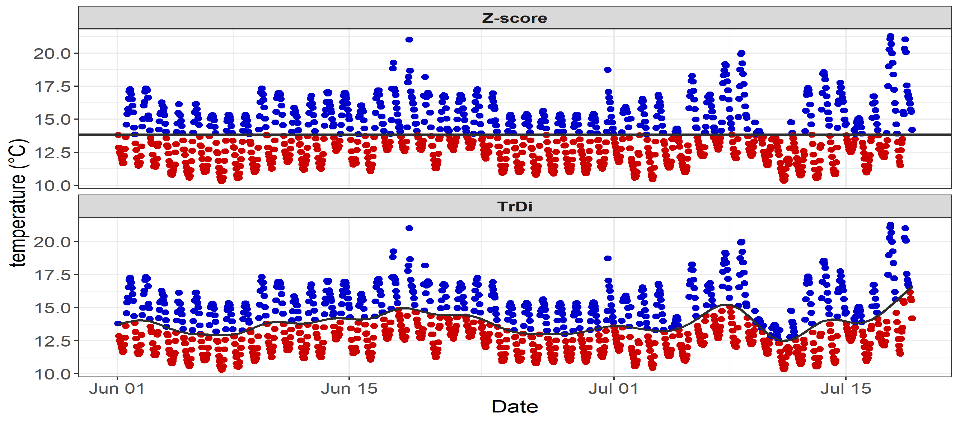


Figure SM21: Loch Lintrathen Upstream, year 2017, standardized temperatures with $Zs$ and ${Tr}_{Di}$ (top 3 row). In the fourth and fifth row are reported water temperature values standardized as positive (**BLUE**) or negative (**RED**) according to $Zs$ and ${Tr}_{Di}$ standardization. The horizontal line in Z-score graph is the mean water temperature during the period, the wiggly line in the ${Tr}_{Di}$ graph is the modelled $Tr$.

1. **Water temperature and water temperature rate of change statistical summary from 1/06 to 30/07.**

Table SM4: Water absolute temperature summary statistics for the period 01 June -30 July (2016 or 2017, see main text for year of sampling of each location). meanT= mean temperature; Mean daily var.= mean daily variability (i.e., maxT-minT), Mean daily max and mean daily min= average daily maximum and minimum respectively; Max, Min=maximum and minimum T in the period, Last exp. = temperature of the last hour before sampling.

| location | MeanT  (sd) °C | Mean daily var. (sd) °C | Mean Daily max (sd) °C | Mean Daily min (sd) °C | Max  °C | Min °C |
| --- | --- | --- | --- | --- | --- | --- |
| Morie Upstream | 13.6 (1.9) | 3.6 (1.4) | 15.5 (1.6) | 11.8 (1.5) | 20.2 | 8.7 |
| Morie downstream | 14.0 (0.9) | 1.8 (0.7) | 15.0  (0.8) | 13.2 (0.8) | 17.1 | 10.9 |
| Glenquey Upstream (2016) | 9.6 (1.0) | 2.9 (0.8) | 11.2 (0.7) | 8.4 (0.4) | 13.0 | 7.6 |
| Glenquey Downstream (2016) | 12.4 (0.9) | 2.0 (0.6) | 13.5 (0.8) | 11.5 (0.7) | 15.0 | 10.2 |
| Glenquey Upstream (2017) | 10.3 (1.0) | 3.0 (0.9) | 12.1 (0.8) | 9.0 (0.4) | 14.7 | 7.8 |
| Glenquey Downstream (2017) | 13.1 (0.9) | 2.7 (0.7) | 14.6 (0.8) | 11.9 (0.5) | 16.6 | 11.1 |
| Rescobie Upstream | 13.8 (0.9) | 1.6 (0.5) | 14.6 (0.9) | 13.0 (0.7) | 17.0 | 11.8 |
| Rescobie Downstream | 16.4 (1.9) | 2.9 (0.8) | 18.0 (1.7) | 15.1 (1.8) | 21.6 | 10.6 |
| Lintrathen Upstrteam | 13.9 (2.0) | 5.5 (1.6) | 16.9 (1.7) | 11.4 (0.7) | 21.3 | 10.3 |
| Lintrathen Downstream | 14.0 (1.8) | 0.9 (0.4) | 14.5 (1.8) | 13.6 (1.8) | 17.5 | 10.7 |

*Table SM5:* ${At}_{r}$*summary statistics for the period 01/06 -30/07. Mean positive* ${At}_{r}$*= mean positive temperature change rate; Mean negative* ${At}_{r}$*= mean negative temperature change rate.*

| location | Mean positive ${At}_{r}$  (±sd) °C/h | Mean negative ${At}_{r}$  (±sd) °C/h |
| --- | --- | --- |
| Morie Upstream | 0.4 (0.4) | -0.3 (0.3) |
| Morie Downstream | 0.3 (0.3) | -0.2 (0.3) |
| Glenquey Upstream (2016) | 0.4 (0.6) | -0.4 (0.5) |
| Glenquey Downstream (2016) | 0.3 (0.4) | -0.3 (0.3) |
| Glenquey Upstream (2017) | 0.5 (0.7) | -0.5 (0.6) |
| Glenquey Downstream (2017) | 0.5 (0.6) | -0.5 (0.5) |
| Rescobie Upstream | 0.2 (0.2) | -0.1 (0.1) |
| Rescobie Downstream | 0.3 (0.3) | -0.2 (0.2) |
| Lintrathen Upstream | 0.6 (0.6) | -0.4 (0.3) |
| Lintrathen Downstream | 0.1 (0.1) | -0.1 (0.1) |

1. **CT_max_: summary and significant differences**

In all the analysis here reported outlier values have been removed prior to estimation of $\overline{{CT}_{max}}$, as described in the main text. A Generalized Linear Model (GLM) in the form

$$CTmax=species+ type+location+\varepsilon$$

was used to establish overall significant differences between CTmax in different groups. $species$ factor refers to the sample species and $type$ is a factor indicate if the sampling location was dammed or natural.

Table SM6: t-ratio and p-value of the comparison between $\overline{{CT}_{max}}$ in dammed and natural samples and between sampled species.

| factors | $\overline{{CT}_{max}}$ ± sd (°C) | t-ratio | p-value |
| --- | --- | --- | --- |
| dam | 34.5±3.4 | 0.28 | 0.7 |
| natural | 34.1±3.9 |  |  |
|  |  |  |  |
| *Serratella ignita* | 29.8 ± 2.2 | -20.8 | <0.0001 |
| *Baetis rhodani* | 35.4 ± 3.0 |  |  |

To establish if subsequent sampling within the same location significantly differs in their mean CT_max_, we implanted the equation separately for each population (i.e., species, site and location)

$$CTmax=sample+\varepsilon$$

Where $sample$ is a factor identifying the timing of the sample. We Estimated the marginal means (EMMs) with the above models to establish the significance of the difference in the means between all the groups listed. The test applied was a T-test adjusted with Sidak method for pairwise comparisons. In Table 7 are reported all the results of the compared samples within each sampling location.

t-ratio: 0.422, p-val: 0.90

t-ratio: -3.143 p-val: 0.0063

t-ratio: 1.278, p-val: 0.41

t-ratio: 3.997, p-val: 0.0003

t-ratio: -1.517 p-val: 0.29

t-ratio: 2.765, p-val: 0.009

t-ratio: -2.498, p-val: 0.016

t-ratio: -2.841, p-val: 0.006

t-ratio: 0.432, p-val: 0.90

t-ratio: 1.609, p-val: 0.25

t-ratio: 2.771, p-val: 0.017

t-ratio: 0.997, p-val: 0.58

t-ratio: 0.041, p-val: 0.99

t-ratio: -3.721, p-val: 0.0009

| **Site** | **year** | **Location** | **type** | **species** | **sample** | $\overline{\boldsymbol{CT}_{\boldsymbol{max}}}\boldsymbol{\pm sd}$ **by sample** |
| --- | --- | --- | --- | --- | --- | --- |
| Morie | 2016 | Upstream | Natural | *B. rhodani* | 1 | 30.8±2.1 |
|  |  |  |  |  | 2 | NA |
|  |  |  |  |  | 3 | 29.1±1.8 |
|  |  | Downstream | Natural |  | 1 | NA |
|  |  |  |  |  | 2 | NA |
|  |  |  |  |  | 3 | 32.6±0.1 |
| Glenquey | 2016 | Upstream | Natural |  | 1 | NA |
|  |  |  |  |  | 2 | 27.8±2.0 |
|  |  |  |  |  | 3 | 29.3±2.4 |
|  |  | Downstream | Dam |  | 1 | NA |
|  |  |  |  |  | 2 | 30.0±1.4 |
|  |  |  |  |  | 3 | 31.1±1.5 |
|  | 2017 | Upstream | Natural | *S. ignita* | 1 | 34.0±2.3 |
|  |  |  |  |  | 2 | 33.7±2.7 |
|  |  |  |  |  | 3 | 32.9±2.6 |
|  |  | Downstream | Dam |  | 1 | 35.4±1.7 |
|  |  |  |  |  | 2 | 33.5±3.5 |
|  |  |  |  |  | 3 | 32.9±2.7 |
| Lintrathen | 2017 | Upstream | Natural |  | 1 | 36.0±3.5 |
|  |  |  |  |  | 2 | 36.0±3.2 |
|  |  |  |  |  | 3 | 38.5±1.4 |
|  |  | Downstream | Dam |  | 1 | 37.5±1.1 |
|  |  |  |  |  | 2 | 35.5±2.8 |
|  |  |  |  |  | 3 | 36.3±3.0 |
| Rescobie | 2017 | Upstream | Natural |  | 1 | 37.0±1.4 |
|  |  |  |  |  | 2 | 36.4±1.8 |
|  |  |  |  |  | 3 | 37.7±1.7 |
|  |  | Downstream | Natural |  | 1 | 35.3±3.3 |
|  |  |  |  |  | 2 | 34.9±3.3 |
|  |  |  |  |  | 3 | 39.1 |

Table SM 7: Mean CTmax and standard deviation for each sample. Refer to Fig. 1 in the main text for the sample size. Site: reference lake; **Location**: upstream or downstream of the reference lake in "site; **Type**: if downstream of a dam or not; $\overline{\boldsymbol{CT}_{\boldsymbol{max}}}\boldsymbol{\pm sd}$ **by sample:** mean CTmax and standard deviation in °C. On the right side are reported for each subsequent sample within sampling location the result form T-test based on EMMs.

1. **Serratella ignita DLNM analysis**

To verify that our models reported in the main text are comparable for single-species comparisons, we implemented for solely *S. ignita* $\overline{{CT}_{max}}$ data the best three models found for the species combined dataset, respectively $Zs$3, ${Tr}_{Di}$3 and ${At}_{r}$3 (Tab. 1 main text). The equation takes the form

$$\left( y_{i,t} \right)=cb+u_{sl}$$

where $y_{i,t}$represents *S. ignita* $\overline{{CT}_{max},}$ and $u_{sl}$ is sampling location as a random effect. Finally, $cb$, the crossbasis part of DLNM, is implemented with cubic regression spline with 3 degrees of freedom in lag and exposure space, including the intercept in the lag dimension.

The performance of the three models reported in Table SM8

Table SM8: model performance for DLNM implemented on solely Serratella ignita data.

| **Model** | $\boldsymbol{f}\left( \boldsymbol{x} \right)$ | $\boldsymbol{w}\left( \boldsymbol{l} \right)$ | **intercept** | **Model performance** | |
| --- | --- | --- | --- | --- | --- |
|  |  |  |  | **AIC** | **ED%** |
| Zs | cr,df=3 | cr,df=3 | Yes | 69.5 | 58.5 |
| *Tr_Di_* | cr,df=3 | cr,df=3 | Yes | 66.6 | 69.9 |
| *At_r_* | cr,df=3 | cr,df=3 | Yes | 57.5 | 87.1 |

Similarly to main analysis, Tr_Di_ and At_r_ models perform better than Zs model. The overall effect surface is similar for each exposure to the one estimated for both *S. ignita* and *B.rhodani* together in terms of shape and magnitude of predicted effect. Nonetheless, the lower number of observations here increase estimation uncertainty: no effects of *Zs* and *Tr_Di_* models are significant, while the At_r_ model is marginally significant for extreme vlaues (Fig. 22).

**
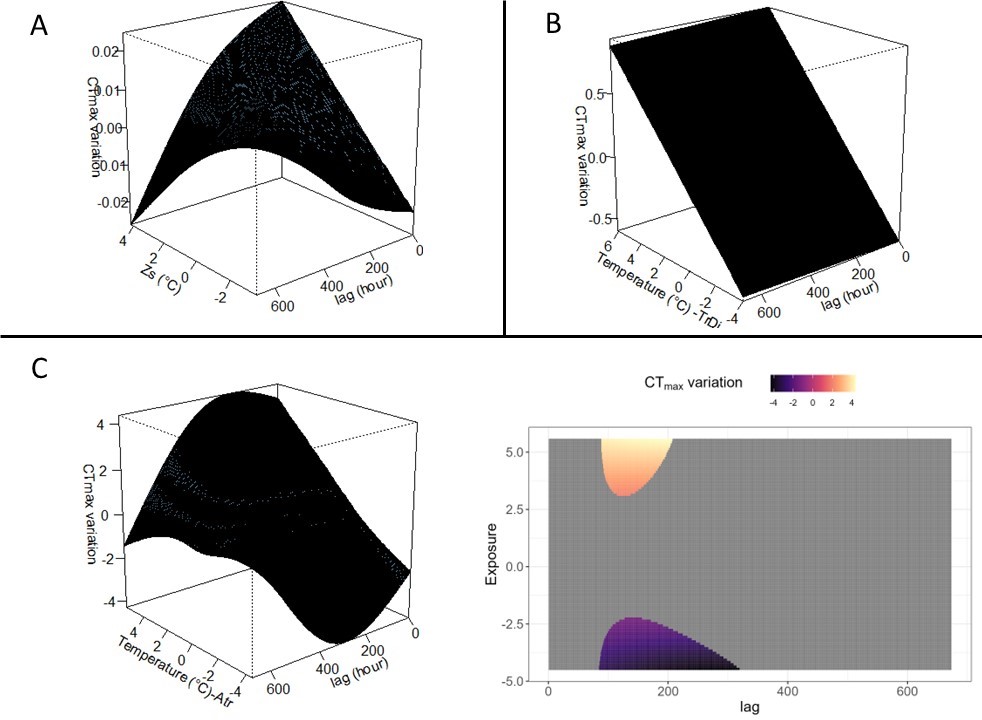
**

Figure SM22: A- Overall surface predicted form Zs model on solely Serratella ignita data, no effects are significant. B Overall surface predicted form Tr_Di_ model on solely Serratella ignita data, with no effects significant. C- Overall surface predicted form At_r_ model on solely Serratella ignita data, with a small portion of the surface marginally significant.

1. **Zs ESTIMATED EFFECT**


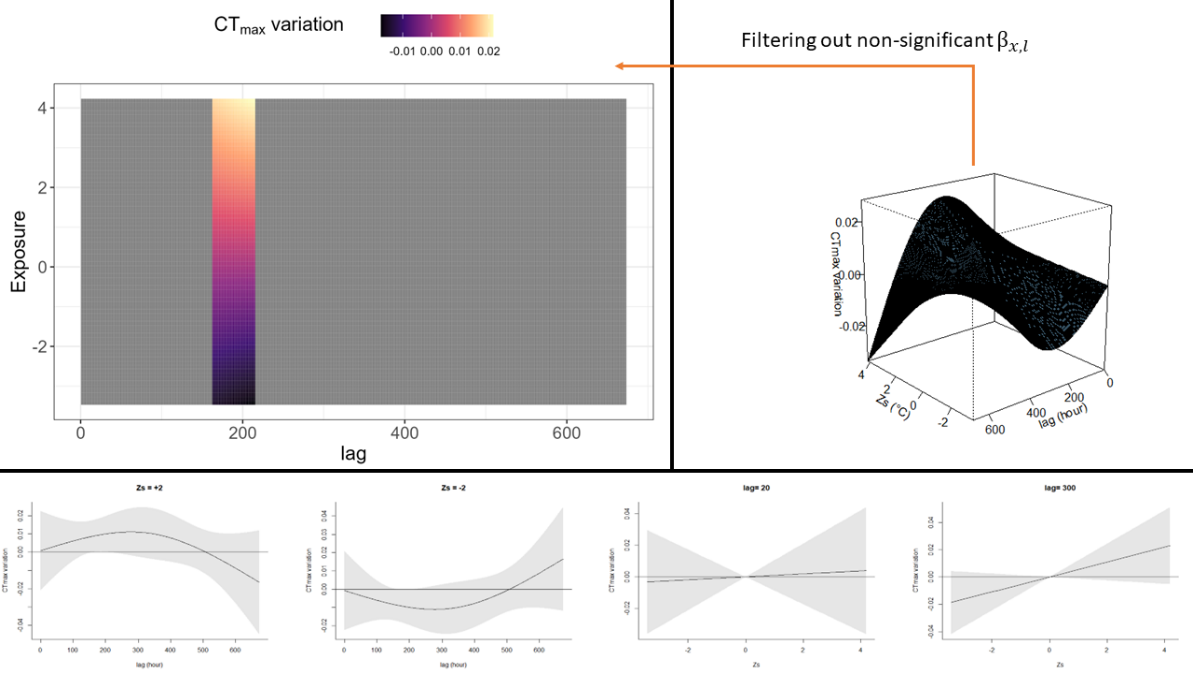


Figure SM23: DLNM plot for effect of Temperature magnitude, Zs standardized, on $\overline{\boldsymbol{CT}_{\boldsymbol{max}}}$ estimated with $\boldsymbol{Zs}$3 model. The overall plot (average effect) shows marginal effects compared to ${Tr}_{Di}$ and ${At}_{r}$. A) 2d heatmap of the overall surface, the grey area corresponds to $\beta_{x,l}$ where the 95% c.i. includes 0. B) default overall plot from dlnm package of the $Zs$ – lag-response surface. C) default plot offered by the “dlnm” package, slicing the surface on the lag and exposure dimension; by way of example, we sliced at lag=20, lag=300, $Zs$= -2 unit sd and $Zs$= +2 unit sd; shading represents 95% c.i.

1. **ONLY MEASURED WATER TEMPERATURE DATA DLNM**

We modelled some portion of the thermal history to account for missing data and allow for a 28 day long thermal history exposure, in order not to further shorten the period of possible effects on CT_max_ (see main paper and SM1). Nonetheless, some of the modelled portions of ther thermal history may have underestimated the thermal variability present in the measured data (see validation plots, figures S2- S11) To ensure the error in the thermal exposure estimation did not causes substantial biases in the estimation of the effects on $\overline{{CT}_{max}}$through the DLNM, we modelled again through DLNM only the samples with a field-measured thermal history. Specifically, we restricted the analysis to a 19 days-long thermal history excluding all the samples with a number of missing days > 9 days (n = 8 sampling events excluded, see Table SM1). In Figure 24 is reported the Tr_Di_ and At_r_ estimated effect and the significant combination of lag and exposures estimated for this reduced dataset.

We found that the overall effect estimation for the measured-only thermal history data is predicted to have a similar shape top the one presented in the main text. In both At_r_ and Tr_Di_ models, the absolute effect in terms of °C is predicted to be higher in magnitude for the full data (main text model), as for the reduced (measured-only thermal history) presented here. This is explained by the fact that the model has been forced to distribute the same effect of environmental temperature on $\overline{{CT}_{max}}$over a shorter period of time (only 19 days rather than 28). Furthermore, despite the fact that the modelled thermal exposures have in some cases underestimated the thermal extremes present in the real exposure history (see figure S2-S11), the result on the measured-only exposure data shows *smaller* impacts of extreme exposure on $\overline{{CT}_{max}}$.

Here, At_r_ effects are largely congruent in terms of significant combination of lag-exposure with the one presented in the main text (Fig. SM24B vs Fig 5 main text). Tr_Di_ effects reported here are estimated to be similar in the overall shape of the main text findings (Fig. SM24A vs. Fig. 4 main text), but the significant area is different with the one found in the main text. As stated above, differences may result from reduced sample sizes, as this analysis is performed over a reduced number of samples as well as days, with a similar effect as the analysis done on solely *Serratella ignita* data (see SM5).

The main potential source of bias induced by the water temperature estimation on the DLNM was related to the effect of the under- and over- estimation of maximum and minimum temperatures from the models, respectively (see SM1); this could have forced the DLNM to allocate the major effects on CT_max_ on the field-measured water temperatures, resulting in a concentration of the effects in the latest part of the timeseries (where there are almost no missing data, see Tab. SM1). Nonetheless, with this analysis we showed that the overall estimated effect, if performed only on measured data, still follows the same shape and interaction between exposure and lag found in the main text. This analysis therefore also demonstrates that the significant lag dimensions from main text results are also not biased by the modelled data.


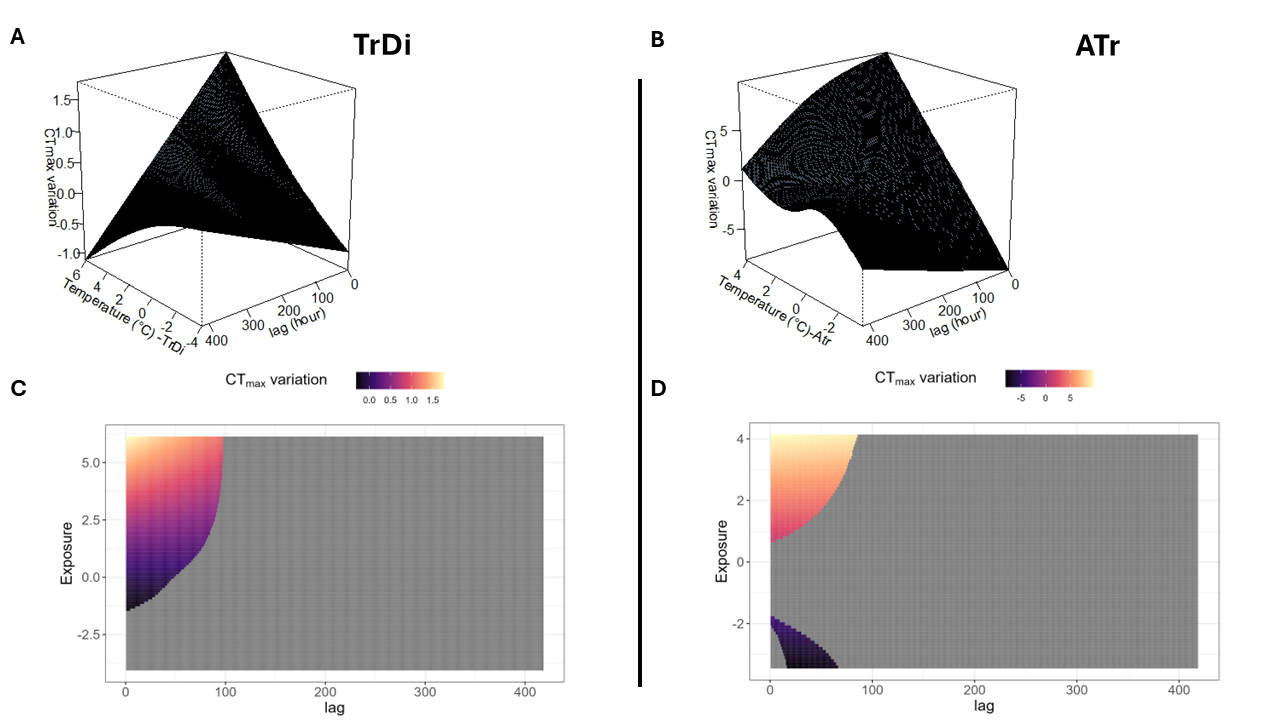


Figure SM24: Overall effect plot and 2D heat map effect of TrDi (A, C) and ATr (B, D) for the samples with only measured thermal history of 19 days.

References

[1] Met Office, “Met Office Integrated Data Archive System (MIDAS) Land and Marine Surface Stations Data (1853-current). NCAS British Atmospheric Data Centre, 07/03/2023.” 2012. [Online]. Available: http://catalogue.ceda.ac.uk/uuid/220a65615218d5c9cc9e4785a3234bd0

[2] S. Wood, *Generalized Additive Models: An Introduction with R, Second Edition*. Chapman and Hall/CRC, 2017. Accessed: Sep. 17, 2020. [Online]. Available: https://www.routledge.com/Generalized-Additive-Models-An-Introduction-with-R-Second-Edition/Wood/p/book/9781498728331

[3] R. V. Lenth, “emmeans: Estimated Marginal Means, aka Least-Squares Means.” 2022. [Online]. Available: https://cran.r-project.org/package=emmeans
